# DNA Metabarcoding Illuminates Seasonal Dietary Pattern and Niche Partitioning by Three Sympatric Herbivores

**DOI:** 10.1002/ece3.71321

**Published:** 2025-04-21

**Authors:** Dandan Wang, Zhiming Cao, Yuqin Liu, Ruofei Li, Ruitao Wu, Wenguo Wu, Wuhua Liu, Xiaolong Hu, Yongtao Xu

**Affiliations:** ^1^ Jiangxi Provincial Key Laboratory of Conservation Biology Jiangxi Agricultural University Nanchang Jiangxi China; ^2^ Taohongling Sika deer National Nature Reserve Pengze Jiangxi China; ^3^ College of Animal Science and Technology Jiangxi Agricultural University Nanchang Jiangxi China

**Keywords:** diet, herbivores, niche partitioning, plant–herbivore interactions, sika deer, sympatric species, trophic niche

## Abstract

Diet composition is among the most critical dimensions of animal ecology, yet seasonal dietary diversity has rarely been investigated in sympatric herbivores. This study used DNA metabarcoding to conduct an analysis of seasonal variations in diet composition and trophic niches for sympatric sika deer, Reeves' muntjac, and Chinese hare in Taohongling National Nature Reserve (TNNR). The results showed that *Smilax china* (11.79%) was the leading food eaten by sika deer in summer, whereas dominated by *Rubus* spp. (36.42%) and *Loropetalum chinense* (25.48%) in winter; *Rubus* spp. accounted for the majority of Reeves' muntjac's diet throughout the year. In comparison, the Chinese hare primarily consumed *Smilax china* from winter to spring but changed to *Poa annua* (10.81%) and *Setaria viridis* (23.05%) in summer and fall. Compared to other seasons, significant differences (Shannon index, *p* < 0.05) occurred in spring and summer, showing higher diversity of food items across the three herbivorous. Nonmetric multidimensional scaling (NMDS) analysis suggested significant partitioning in the food items of Chinese hares compared to the two ruminants. Both sika deer and Reeves' muntjac occupied a wider niche breadth and dietary diversity in summer, reflecting generalised feeding habits (Sd: Ba = 0.06; Rm: Ba = 0.04) and lower in fall (Ba = 0.01) with stronger selectivity and specialization, which was consistent with the optimal foraging theory. Notably, no significant difference was indicated in seasonal niche breadth for Chinese hare (*p* > 0.05). The niche overlap indices were 0.989 (fall) and 0.831 (winter) between sika deer and Reeves' muntjac, indicating a higher dietary similarity and overlap. However, differences in foraging plant taxa and abundance ratios may facilitate dietary niche partitioning. The diet of herbivores reflected plant–herbivore interactions and seasonal diet differences were correlated with feeding strategies, which facilitate coexistence and reduce competition of co‐occurring species in the food dimension.

## Graphical abstract

In our research, we found extensive trophic niche overlap based on the trnL metabarcoding sequencing, while dietary partitioning may facilitate species coexistence. Our results provide new insights into seasonal diets and inter‐species relationships for sympatric herbivores.

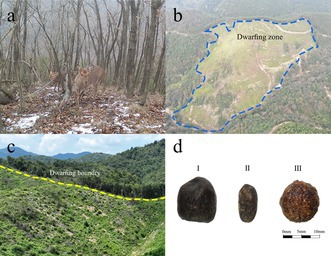

## 1 Introduction

Food provides the essential energy and nutrients required for animal life, making it a crucial factor for the survival and development of wildlife populations (Zhang et al. 2020). Due to global climate change, excessive exploitation, and other factors, wildlife habitats have shrunk and become fragmented, followed by the decline of available food resources (Kowalczyk et al. 2011), which drives and accelerates the extinction risk of herbivores (Atwood et al. 2020). To withstand environmental change, wild populations often exhibit dietary flexibility (Hutchinson et al. 2022). The mixed feeders (eating both grasses and trees/shrubs) of savanna ungulates, which primarily graze in the wet season, switch to browsing (eating predominantly trees and shrubs) in the dry season as grass biomass and nutrition decline (Codron et al. 2007; Abraham et al. 2019). Thus, herbivores instead alter their diets seasonally in response to local changes in resource quality and availability (Kartzinel and Pringle 2020). Dietary composition provides valuable insights into herbivores' foraging ecology, plant–herbivore interactions, as well as the availability of resources (Owen‐Smith et al. 2010). Plants are influenced by phenological periods and seasons; the variations in food resources are particularly impactful for herbivores, as they are highly dependent on specific plant resources. However, most studies on herbivores feeding habits have focused mainly on foraging plant descriptions without addressing seasonal dietary patterns.

The overlap and partitioning in diet composition modulates competition intra‐ and interspecies (Tinker et al. 2008). The features of an ecological niche include eating, competition, predation, and defense (Kearney et al. 2010). Based on the niche theory, the trophic niche is the result of the long‐term evolution of species. It can reflect the pressures wildlife experience in habitats and helps to gain a deeper understanding of the interspecific relationships within a community or ecosystem (Lei et al. 2024; Xiao et al. 2023). The competitive exclusion principle states that in the case of coexisting organisms sharing the same resources, competition will eventually occur once the densities of the coexisting species and/or the availability of their resources have reached their respective carrying capacities (Metzger et al. 2009; Gause 2019). Intense competition occurs between *Cervus wallichii* and *Cervus albirostris* in the grass‐growing season, as reflected by the diet niche breadth and overlap index (Lv et al. 2020). Species coexistence theory emphasizes that niche partitioning plays a central role in the coexistence of sympatric species (Schaller 2000). Owing to long‐term evolutionary processes, co‐occurring species of similar diets can partition the food axis, among other niches, which allows them to reduce resource competition (Prins et al. 2006; Yao et al. 2021; Lamichhane et al. 2025), thereby facilitating species coexistence and community stability.

Precise identification and diet assessment are critical for the effective conservation and management of wildlife. DNA metabarcoding techniques, which utilize short DNA fragments, refer to the automated identification of multiple species from a single bulk sample containing all organisms or from a single environmental sample containing degraded DNA (soil, water, feces, etc.; Taberlet et al. 2012; Valentini et al. 2009; Barco et al. 2016; Hambäck et al. 2016). With the advancement of high‐throughput sequencing technology, DNA metabarcoding has emerged as an effective tool applied to address various aspects of wildlife diet (Pompanon et al. 2012), that is, predator–prey or producer–consumer interactions and resource partitioning (Srilopan et al. 2025). This approach has been widely applied to species such as Père David's deer ( *Elaphurus davidianus* ), European bison ( *Bison bonasus* ), Red Deer ( *Cervus elaphus* ), and sika deer ( *Cervus nippon* ) (Lin et al. 2024; Hartvig et al. 2021; Ratkiewicz et al. 2024; Nakahama et al. 2021). Kartzinel et al. (2015) quantified the diet composition of seven large mammalian herbivore species in the semiarid African savanna, indicating enormous potential in biodiversity research to obtain much‐needed information on the resource and niche requirements of herbivores.

**FIGURE 1:**
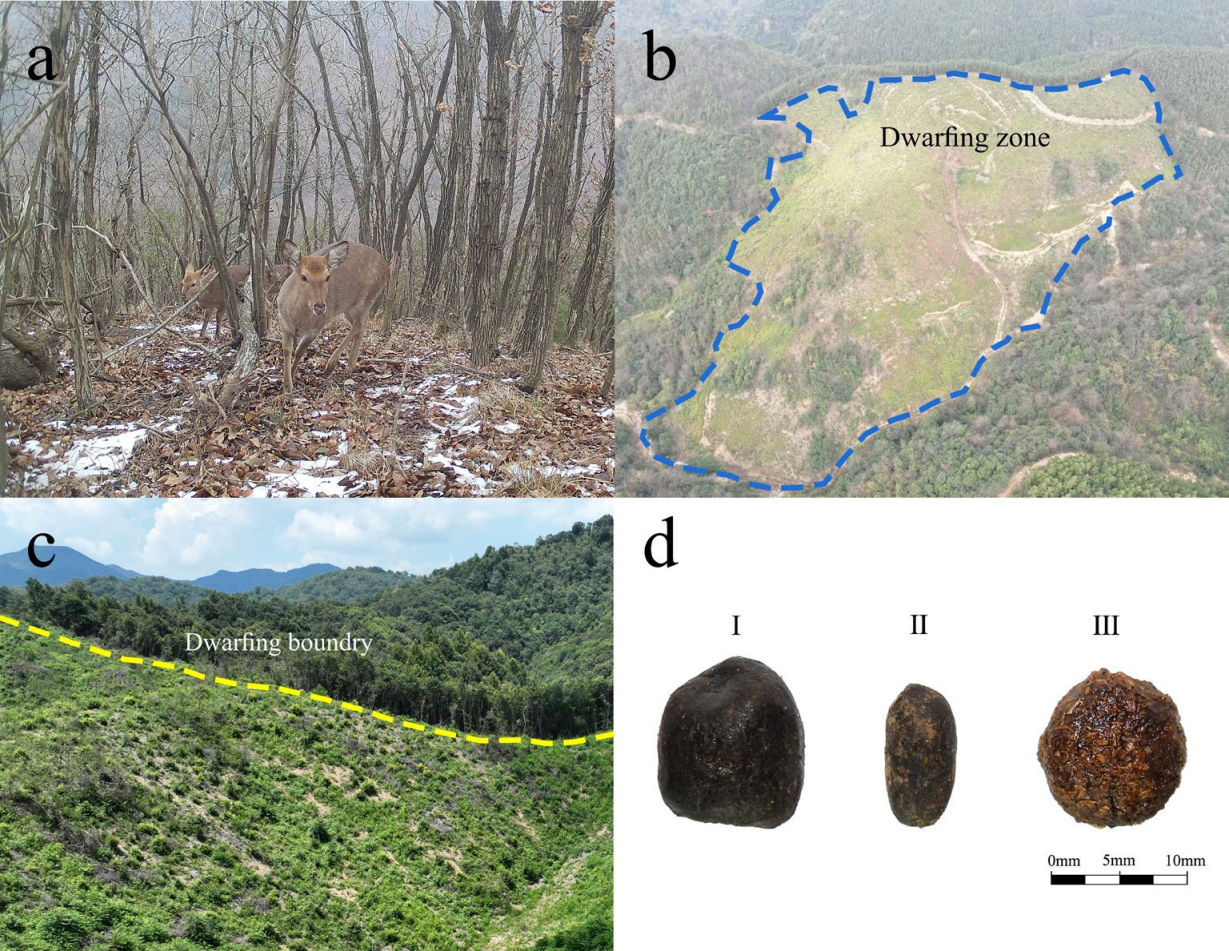
Ecological photographs in Taohongling Sika Deer National Nature Reserve (TNNR). (a) The reduced understory forage due to vegetation succession. (b) An aerial view of the artificial vegetation dwarfing experimental plot within the TNNR. (c) Comparison between the dwarfed and nondwarfed experimental areas. (d) Morphological and dimensional characteristics of fecal pellets for three sympatric herbivores (I: sika deer, II: Reeves' muntjac, and III: Chinese hare).

The sika deer is a medium‐sized member of the Cervidae family (order Cetartiodactyla) and has been classified as a National Class I protected animal in China (Xu et al. 2024). Historically, sika deer have spread across East Asia with 13 recorded subspecies (Whitehead 1972). However, the subspecies found in Shanxi ( *Cervus nippon grassianus* ), North China (*Cervus nippon mandarinus*), and Taiwan (*Cervus nippon taiouanus*) became extinct successively by the 1960s, after both natural and artificial disturbances. At present, only three subspecies from Northeast China (*Cervus nippon hortulorum*), Sichuan (*Cervus nippon sinchuanicus*), and East China (*Cervus nippon kopschi*) still exist in China (Wei 2022; Guo and Zheng 2000). Because the distribution areas are small and isolated, communication and gene flow between populations are difficult (Su et al. 2023). The Taohongling Sika Deer National Nature Reserve (TNNR) is the largest habitat for East Chinese sika deer (Wang, Hu, et al. 2023). Over the years, carrying capacity and reduced understory forage have been observed due to vegetation succession (Figure 1a; Li et al. 2024). Sika deer are frequently observed escaping from the reserve to forage in surrounding agricultural fields, orchards, and nurseries. To enhance habitat suitability for sika deer, habitat dwarfing measures have been implemented within the reserve (Figure 1b,c).

As the main sympatric ungulate of sika deer in the TNNR, the Reeves' muntjac is widely distributed and serves as a key prey for various carnivores (i.e., *Panthera pardus* ; *Cuon alpinus* ), playing an essential ecological role in the food web of forest ecosystems (Jiang 2009; Chen et al. 2022). Field investigation and monitoring revealed that the relative abundance of Reeves' muntjac (camera traps, vocalizations, and fecal analysis) was considerably higher than that of the sika deer in TNNR (Kong et al. 2024). In addition, the Chinese hare ( *Lepus sinensis* ), a grass‐eating species endemic to China, also coexists with the two ruminants (Wei 2022). The three herbivores have distributed sympatrically in the TNNR for decades, but whether any variations and commonalities exist in their diet is yet least understood.

Knowledge of what wild herbivores eat, how their dietary pattern varies over the season, and whether sympatric species compete for food resources is critical to understanding their ecology and interspecific interaction. This study aims to improve our understanding of the dietary habits of sympatric herbivores that inhabit TNNR, specifically exploring the seasonal diet pattern using DNA metabarcoding. The objectives of this study were to assess the dietary profile of three sympatric herbivores in terms of (1) the seasonal diet composition of three species and (2) the extent of resource partitioning and interspecific trophic niches. By examining the diets of these three species and quantifying their diet overlap, this study will provide insights into molecular diet and interspecific niche studies in co‐occurring herbivores. Additionally, it would be significant to population conservation and management of sika deer and biodiversity monitoring.

## 2 Materials and Methods

### 2.1 Study Area and Sample Collection

**FIGURE 2:**
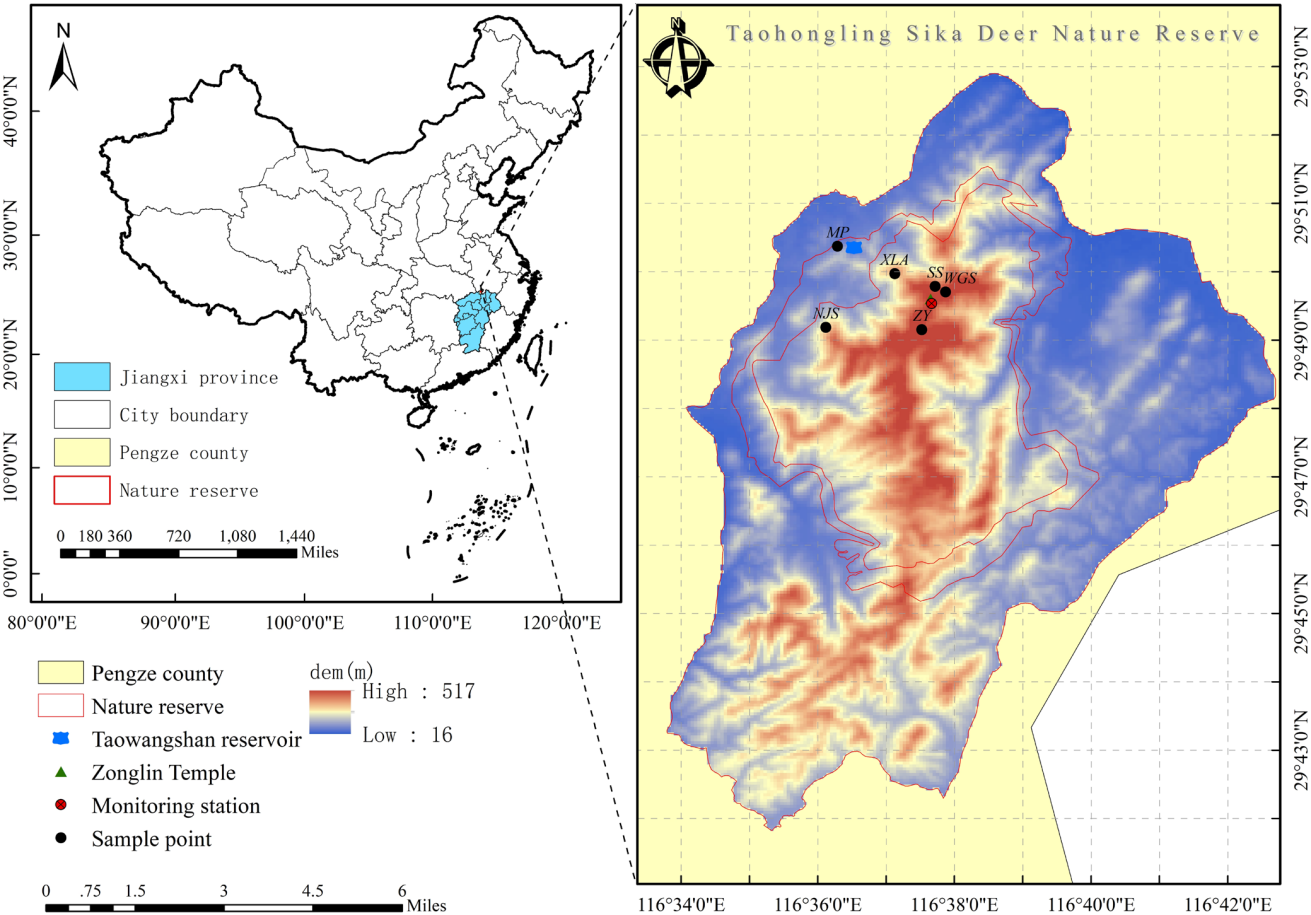
Fecal sample sites of three sympatric species at Taohongling Sika Deer Nature Reserve (MP: nursery bases; SS: fir forests; NJS: NieJiashan; XLA: XianLingAn; WGS: WuGuiShi; ZY: Bamboo Garden).

The TNNR is located on the southern bank of the lower reaches of the Yangtze River in Pengze County, Jiangxi Province, China. The reserve was set aside to preserve the wild flora and fauna in the northern subtropical zone. The reserve has an area of 12,500 ha, of which the core zone is 2670 ha, the buffer zone is 1830 ha, and the experimental zone is 8000 ha. It was upgraded to a national‐level nature reserve in 2001 to protect the East China sika deer. The TNNR is located in a climatic zone that transitions from subtropical to mid‐subtropical and experiences a subtropical monsoon climate with transitional characteristics and four distinct seasons (Wang, Hu, et al. 2023). Moreover, the terrain of the reserve is predominantly composed of low mountains and hills, with altitude gradients ranging from 100 to 500 m (Wang et al. 2021). Zonal vegetation consists of evergreen deciduous broad‐leaved forests, coniferous forests, shrublands, and bamboo. As the central warm‐temperate plantation coniferous forest, Chinese fir ( *Cunninghamia lanceolata* ) has high density and sparse undergrowth vegetation (Jiang 2009). Sampling sites primarily focused on nursery bases, XianLingAn, Fir forests, WuGuiShi, NieJiashan, and Bamboo Gardens, all of which exhibited frequent animal activity, and three to five transects were established at each sampling point (Figure 2). To avoid or reduce sample repetition from the same individuals, we considered the sampling distance (50–80 m) and fecal morphology (size). The four seasons were defined as winter (December–February), spring (March–May), summer (June–August), and autumn (September–November). A total of 360 fecal samples, including sika deer, Reeves' muntjac, and Chinese hare (with 30 samples each season) from 2022 to 2023, were collected and stored at −80°C; then species were identified by molecular and morphological analyses of fecal samples (Figure 1d).

### 2.2 DNA Extraction and Fragments Amplification by PCR

Host and plant DNA was extracted using a Stool DNA Isolation Kit (FORE GENE, Chengdu, and TIANGEN, Beijing, respectively) according to the manufacturer's guidelines. For each round of DNA extraction and PCR, negative controls were used to monitor for contamination. In this study, we scraped the external surface of each fecal sample to extract DNA from the host species, which is a result of the sloughing of cells from the digestive tract (Hou et al. 2021). Amplification of a partial sequence of the mitochondrial 16S ribosomal RNA (16S rRNA) gene was performed using the forward primer F (5′‐GAGAAGACCCTATGGAGC‐3′) and reverse primer R (5′‐ATAGAAACCGACCTGGAT‐3′), which can unambiguously assign sika deer and other coexisting species (Xiong et al. 2016); the resulting fragment was approximately 357–383 bp (accession number: PRJNA1228627) in length. PCR reactions of 16S rRNA gene were performed in a total volume of 40 μL consisting of 20 μL of 2xT8 High‐Fidelity Master Mix, 2 μL of each of the primers (10 μM), 1 μL DNA, and 15 μL of ddH_2_O was used. PCR was performed as follows: initial denaturation was performed at 98°C for 2 min, followed by 37 cycles of denaturation at 98°C for 10 s, annealing at 51°C for 15 s, and elongation at 72°C for 15 s. Final extension was performed at 72°C for 5 min and storage at 4°C. Amplicons were separated on a 1% agarose gel stained with loading buffer (TSSJ‐20, Tsingke Biotechnology Co. Ltd., Beijing). Sanger DNA sequencing was performed on an automated DNA sequencer ABI3730‐XL (Applied Biosystems, USA). The obtained DNA sequences were aligned with the reference sequences in the GenBank database by the nucleotide BLASTn (https://blast.ncbi.nlm.nih.gov) program.

The P6 loop region of the trnL (UAA) intron was amplified with universal primers g (5′‐GGGCAATCCTGAGCCAA‐3′) and h (5′‐CCATTGAGTCTCTGCACCT ATC‐3′) using a thermocycler PCR system (Gene Amp 9700, ABI, USA). PCR amplifications were carried out in a total volume of 25 μL, including 12.5 μL PCR mix (Tiangen, Beijing, China), 1 μL DNA, 1 μL of each primer (final concentration of each primer is 10 μM), and 9.5 μL H_2_O. The reaction conditions were as follows: denaturation at 95°C for 3 min, followed by 35 cycles at 95°C for 30 s, 56°C for 30 s, and 72°C for 45 s, with a final 10 min at 72°C and storage at 4°C for 10 h. The PCR products were detected using agarose gel electrophoresis and sequenced by Shanghai Personal Biotechnology Co. Ltd. After the individual quantification step, amplicons were pooled in equal amounts, and the 5′ end of each primer was tagged by barcode (adapters), allowing uniquely tagged PCR products. Then, the pair‐end 2 × 250 bp sequencing (next‐generation sequencing, NGS) was performed using the Illumina NovaSeq 6000 platform in the PE‐250 mode (Illumina, CA, USA). Libraries were sequenced using Illumina NovaSeq 6000 SP Reagent Kit (500 cycles) at Shanghai Personal Biotechnology Co. Ltd. (Shanghai, China).

### 2.3 Bioinformatics and Statistical Analysis

From the FASTQ files obtained from NGS, primer sequences were trimmed using the “cutadapt” plugin, and unmatched primer sequences were removed. Reads were merged using the “*fastq_mergepairs*” plugin within the Vsearch (v2.13.4_linux_x86_64) (Rognes et al. 2016). Reads were quality controlled using the “*fastq_filter*,” “*derep_fulllength*,” and “*uchime_denovo*” methods within the Vsearch plugin. In order to obtain operational taxonomic unit (OTUs) relative abundance estimates, amplicon sequences from each sample were then clustered to each OTUs at the same similarity threshold (~97%) (Johnson et al. 2019), utilizing the “*cluster_size*” plugin. Finally, the taxonomy of each OTU was assigned using BLAST against the NT sequence database (“ftp://ftp.ncbi.nih.gov/blast/db/”) and annotation information was obtained using the “*brocc.py*” script based on the references database using local reference libraries (162 plant species from the TNNR; Appendix A: Text A1; GenBank ID: PP081756–PP081917) as a supplementary resource. OTUs that matched two or more taxa were assigned to a higher taxonomic level, which included all taxa. We further converted the read abundance data to relative read abundance (RRA) for the OTUs obtained from each sample (Deagle et al. 2019).

Alpha diversity is a fundamental metric in biodiversity research used to evaluate species diversity within a group (Whittaker 1972). For an estimation of species richness and evenness, which are related to the number of observed OTUs, the Chao1, Pielou's index, and Simpson's indices were calculated for all samples (Chao 1984; Simpson 1949; Schloss et al. 2011). We quantified the sequencing coverage using Good's index (Good 1953). Furthermore, the Shannon–Wiener diversity index was calculated to explore the OTUs and dietary diversity of each species (Shannon 1948). We applied Kruskal–Wallis tests to assess significant differences (*p* value) among different groups after obtaining the overall alpha diversity indices (Kruskal and Wallis 1952). Significant differences between different groups were visualized in QIIME2. Then, we used rarefaction curves to evaluate the sufficiency of sequencing depth and sampling size. Patterns of diet composition and dietary overlap in the sika deer, Reeves' muntjac, and Chinese hare were visualized in a two‐dimensional space using nonmetric multidimensional scaling (NMDS) plots based on Bray–Curtis dissimilarity. To confirm the relationships between sympatric species, we calculated Levins's (1968) and Pianka's index (Pianka 1973) for each herbivore based on RRA values. The dietary breadth (B) of each species was quantified using Levin's index according to the following formula:

(1) $$B=1/\sum_{i=1}^{S}{P_{i}}^{2}$$

where *B* is the Levin's index of niche breadth, *P*_*i*_ is the proportion of food category i out of all foods, and *S* is the total number of prey categories. Hurlbert's formula (Hou et al. 2021) was applied to standardize the trophic niche measure as follows:

(2) $$B_{a}=\left(B-1\right)/\left(S-1\right)$$

where *B*_*a*_ is the standardized trophic niche breadth and the *B*_*a*_ values range from 0 (minimum diet breadth) to 1 (maximum diet breadth). The dietary overlap index of each species (*Q*_*jk*_) was calculated using Pianka's index with the following formula:

(3) $$Q_{\mathrm{jk}}=\frac{\sum_{i=1}^{s}P_{\mathrm{ij}}P_{\mathrm{ik}}}{\sqrt{\sum_{i=1}^{s}{P_{\mathrm{ij}}}^{2}\sum_{i=1}^{s}{P_{\mathrm{ik}}}^{2}}}$$

where *Q*_*jk*_ is Pianka's niche overlap index between species *j* and *k*, *P*_*ij*_ is the proportion of resource *i* out of all the resources used by species *j*, and *P*_*ik*_ is the proportion of resource *i* out of all the resources used by species *k*. The values ranged from 0 (no food item in common) to 1 (complete overlap in resource use). *Q* > 0.6 indicates a significant overlap (Wallace 1981).

## 3 Results

### 3.1 OTUs Statistics of trnL Sequencing

**FIGURE 3:**
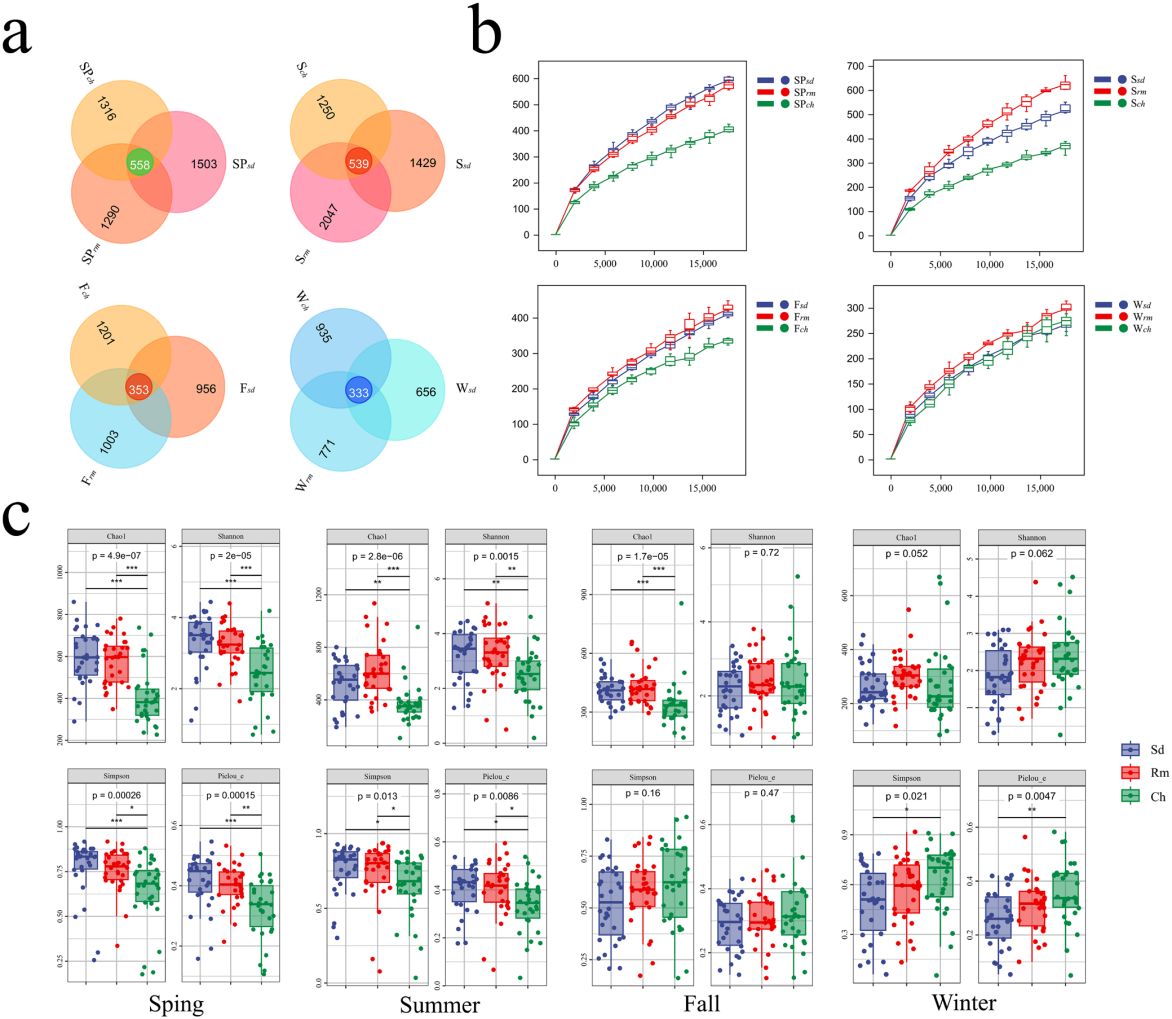
(a) Venn analysis of OTUs of three herbivores in four seasons. (b) Rarefaction curves (*X*‐axis: rarefaction depth; *Y*‐axis: the median value of Shannon indices calculated 10 times). As the sequencing depth increased, the number of OTUs also increased. (c) Boxplots of alpha diversity in four seasons. The horizontal line inside the boxes defines the median; circles outside the boxes indicate outliers. (Sd, Rm, and Ch represent the sika deer, Reeve's muntjac, and Chinese hare, respectively; Sp, S, F, and W mean spring, summer, fall, and winter, respectively).

In metabarcoding analyses, the taxonomic assignment is typically achieved by matching DNA sequences to the reference DNA sequences library (Edgar 2018). The amplicons were separated into 21,163 OTUs (trnL‐P6 sequences) at a 97% cutoff level using high‐throughput sequencing, with sequence lengths ranging from 32 to 407 bp. The common and specific OTUs across the three sample sets were identified to determine compositional similarities and differences at the OTU level. Excluding unclassified OTU sequences, OTUs were present in all three herbivores across all seasons. Overlap was greatest in spring and summer, with 558 and 539 OTUs, respectively. During fall and winter, the three species shared 353 and 333 OTUs for food items. The number of specific OTUs found per species across all seasons ranged from 2047 to 771 in Reeves' muntjac, 1316 to 935 in Chinese hare, and 1503 to 656 in Sika deer (Figure 3a).

### 3.2 Alpha Diversity

The alpha diversity index was associated with the rarefaction depth of the OTUs. A rarefaction curve was drawn by randomly sampling a specific number of sequences from each sample to determine the variation in alpha diversity with increasing rarefaction depth. The rarefaction curves plateaued in most cases as the number of sequencing reads increased (Figure 3b), indicating that the sequencing depth was sufficient to capture the majority of OTUs present. This further confirms that an adequate number of fecal samples were collected. At the interspecies level, significant differences in alpha diversity were observed among the three species (*p* < 0.05) in spring and summer. The Chao1 index was highest for sika deer (~599.7), followed by Reeves' muntjac (~578.82) and Chinese hare (~407.15). The Shannon and Simpson indices also indicated the highest diversity in sika deer. In fall and winter, the Shannon indices were not significantly different (*p* > 0.05) among the three herbivores (Figure 3c). All samples had a Good's coverage index above 99%, implying that sequencing provided adequate coverage of diet diversity (Table A1).

### 3.3 Seasonal Changes of Diet

#### 3.3.1 Sika Deer

**FIGURE 4:**
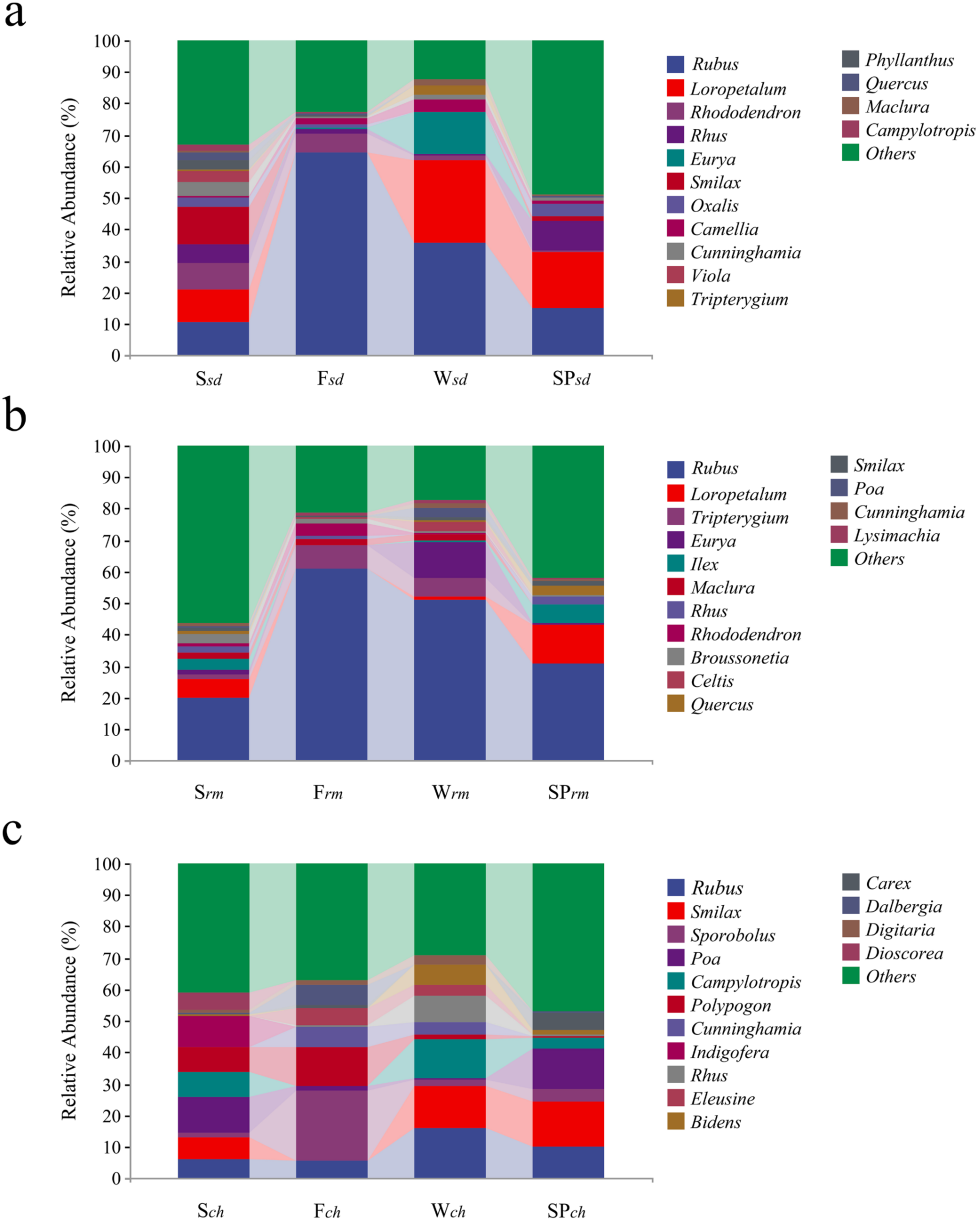
The top 10 genera of foraging plants consumed by three herbivores across different seasons: (a) Sika deer, (b) Reeves' muntjac, and (c) Chinese hare.

The RRA, as a semiquantitative surrogate for the true diet, provides a more accurate view of species' diet to summarize the dietary data (Deagle et al. 2019; Hou et al. 2021). We detected 263 food plant taxa from 115 families of sika deer throughout the year. From spring to winter, 168, 174, 163, and 130 species were identified, respectively. Among them, the genera *Loropetalum* (~17.83%) and *Rubus* (~15.44%) had relatively high proportions in spring, whereas the other categories accounted for only approximately 10% or less. In summer, *Smilax* (~11.80%) was most abundant, followed by *Rubus* (~10.83%), *Loropetalum* (~9.91%), *Rhododendron* (~8.95%), and *Pistacia* (~5.20%). The main genus was *Rubus* in both fall (~64.10%) and winter (~36.49%). At the species level, sika deer foraged on *Loropetalum chinense* (RRA = 17.83%) and *Rubus* spp. (15.20%) in spring and preferred *Smilax china* (11.79%) in summer. In winter, the sika deer reduced their utilization of *Rubus* spp. (36.42%) compared to the fall season (63.95%) and increased their grazing on *Loropetalum chinense* (25.48%) (Figure 4a; Table A2).

#### 3.3.2 Reeves' Muntjac

Throughout the year, 265 forages belonging to 258 genera and 120 families were fed to Reeves' muntjac. In spring, 166 plant species were consumed by Reeves' muntjac, with the genus *Rubus* (~31.81%) and *Loropetalum* (~12.30%) being consumed by Reeves' muntjac. Summer *Rubus* (~20.74%) was the preferred species, followed by *Neolitsea* (~14.01%). In the fall, *Rubus* was still the dominant genus, accounting for 61.40%. During winter, Reeves' muntjac relies on 135 plant species for food; at the family level, Rosaceae and Pentaphylacaceae constitute 63.42% of Reeves' muntjac's diet; among these, the genus *Rubus* accounted for 51.77% and *Eurya* accounted for 11.01%. At the species level, *Rubus* spp. constituted the largest proportion from spring to winter at 31.58%, 20.44%, 61.01%, and 51.77% (Figure 4b; Table A3). The diet compositions of the Reeves' muntjac and sika deer were similar but had seasonal differences in abundance.

#### 3.3.3 Chinese Hare

In spring, Chinese hares primarily consumed plants from *Smilax* (16.95%) and *Poa* (11.23%). In summer, their diet mainly comprised *Poa* (10.81%), *Indigofera* (10.31%), and *Lespedeza* (8.30%). The predominant genera were *Setaria* (23.05%) and *Calamagrostis* (11.09%) in the fall, followed by *Cunninghamia* (6.88%), *Pueraria* (6.05%), and *Dalbergia* (5.53%). During winter, Chinese hares mainly consumed plants from the *Rubus* (15.81%) and the *Smilax* (15.58%), along with *Rhus* (10.64%), *Campylotropis* (9.52%), *Bidens* (5.37%), and *Hedyotis* (5.02%). However, there were no differences in the relative abundances of the predominant genera (*Smilax*) between winter and spring (Figure 4c; Table A4). At the species level, the dominant plants included *Smilax china* , *Rhus chinensis* , *Scleromitrion diffusum*, and *Setaria viridis* , indicating differences in forage consumption compared with Reeves' muntjac and sika deer.

### 3.4 Differences of Foraging Taxa and Intergroup

**FIGURE 5:**
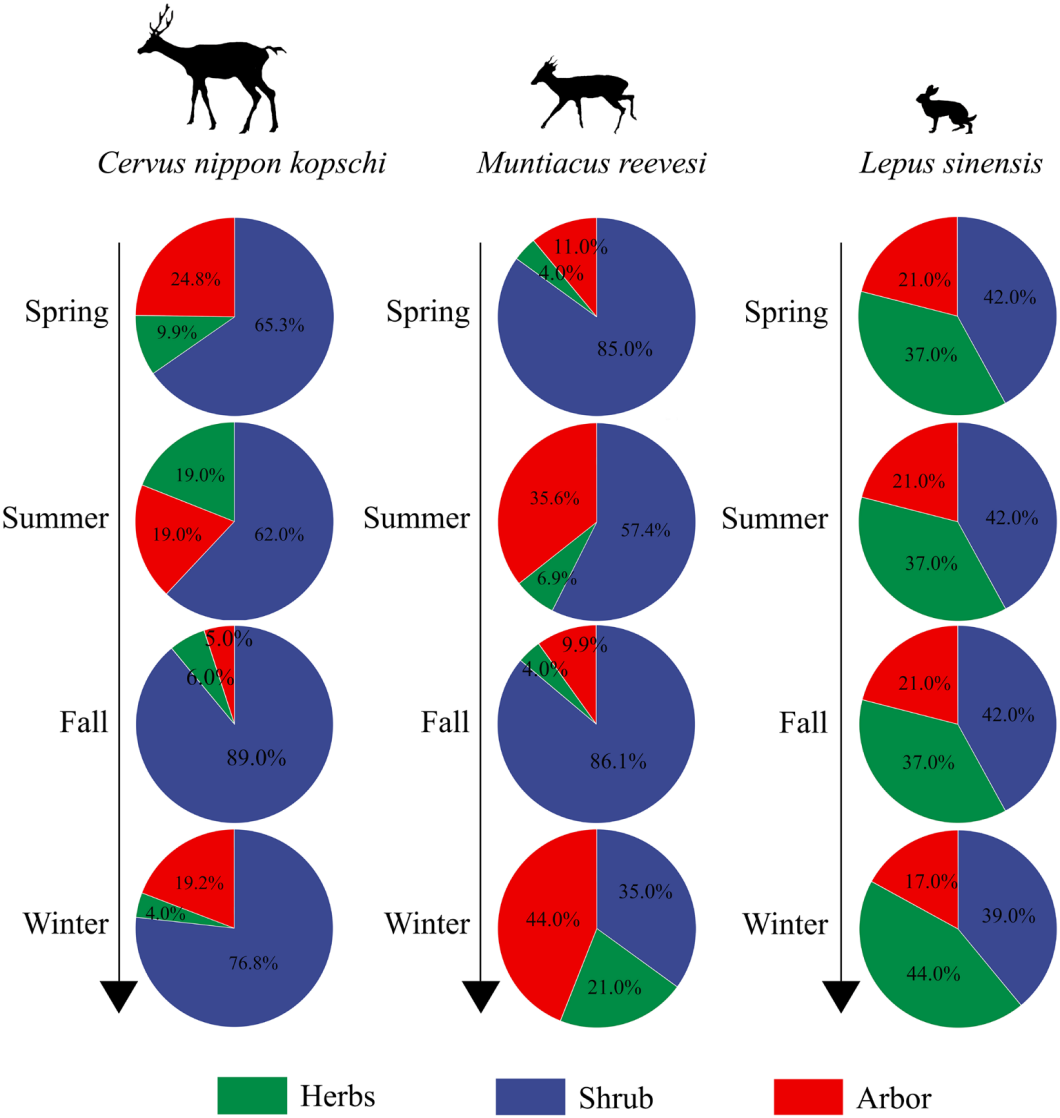
Distribution of plant taxa among three herbivores. Pie diagrams showed the percentage of herbaceous plants, shrubs, and arbors in seasonal change.

Sika deer prefer shrubs and shrub meadows with less shrub‐covered habitats (Fu et al. 2006). Consistent with previous studies, we found a higher preference for shrubs in sika deer, with consumption proportions of 65.3%, 62.0%, 89.0%, and 76.8% across the four seasons, based on the foraging taxonomic group. Due to temporal and spatial variations in plant availability, the proportion of herbaceous plants decreased from 19.0% (summer) to 4.0% (winter). For Reeves' muntjac, the proportion of shrub consumption varied with the seasons, with percentages of 85.0%, 57.4%, 86.1%, and 35.0%, respectively. Reeves' muntjac showed a similar consumption percentage of herbaceous, shrub, and woody plants with sika deer in summer and fall. In winter, the proportion of herbs and arbors increased, and the most remarkable change was the decrease in the shrub category from 86.1% in the fall to 35.0% (Figure 5). However, there were no differences in the foraging taxa of Chinese hares between seasons.

**FIGURE 6:**
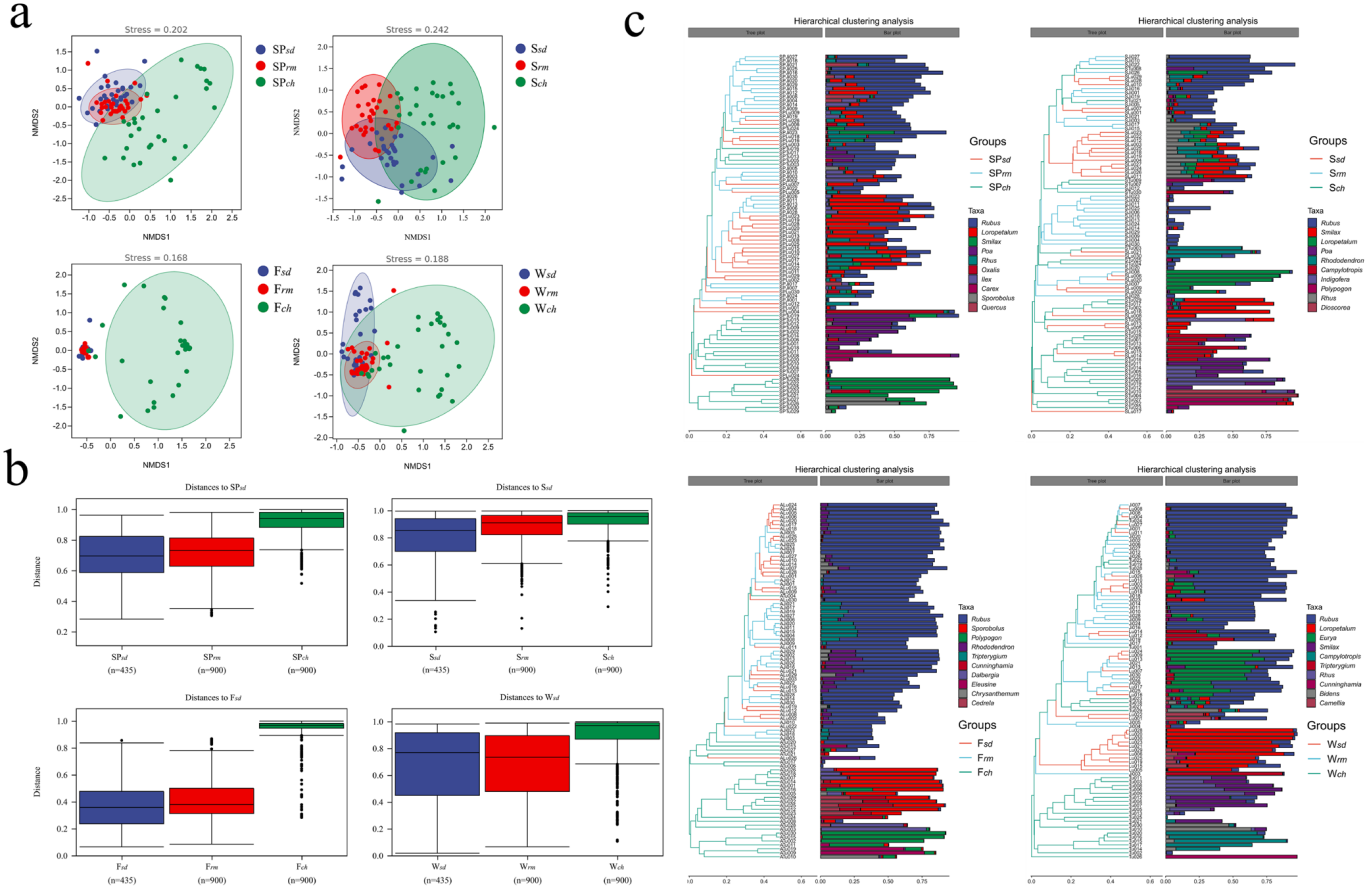
(a) The results of nonmetric multidimensional scaling (NMDS) ordination for three sympatric herbivores based on Bray–Curtis dissimilarity (OTU level). (b) Intergroup difference analysis. Boxplots show the distances between two samples in the sika deer, Reeves' muntjac, and Chinese hare groups in different seasons. (c) Hierarchical clustering diagram. Analysis of the hierarchical clustering tree diagram and the stacked bar diagram of the top 10 genera in abundance.

NMDS classifies, locates, and analyzes research samples in a multidimensional space based on the OTU level. The results indicated that all values showed a high degree of overlap between sika deer and Reeves' muntjac compared to Chinese hares in all four seasons. The overlap between sika deer and Reeves' muntjac was highest in the fall and lowest in summer (Figure 6a). The intergroup difference analysis showed a difference between the intragroup and intergroup sample distances. With different forage compositions, the difference between intragroups should be smaller than those between intergroups. Only in winter, the intergroup distance was slightly lower than the intragroup distance between sika deer and Reeves' muntjac (Figure 6b), suggesting greater differences and richness in the forages consumed within the sika deer group. The seasonal intra‐ and intergroup differences among the three herbivores revealed that the distances between sika deer and Reeves' muntjac were smaller than those of the Chinese hare.

To visualize the similarity between samples, a hierarchical clustering analysis was performed using a stacked bar and clustering tree, providing an intuitive depiction of the species present in each sample. *Rubus* displayed the highest abundance in the food composition of these herbivores, particularly in fall and winter. The clustering tree demonstrated a high level of similarity in the forages consumed by sika deer and Reeves' muntjac across seasons, although both markedly differed from the Chinese hare.

### 3.5 Trophic Niche Breadth and Overlap

**FIGURE 7:**
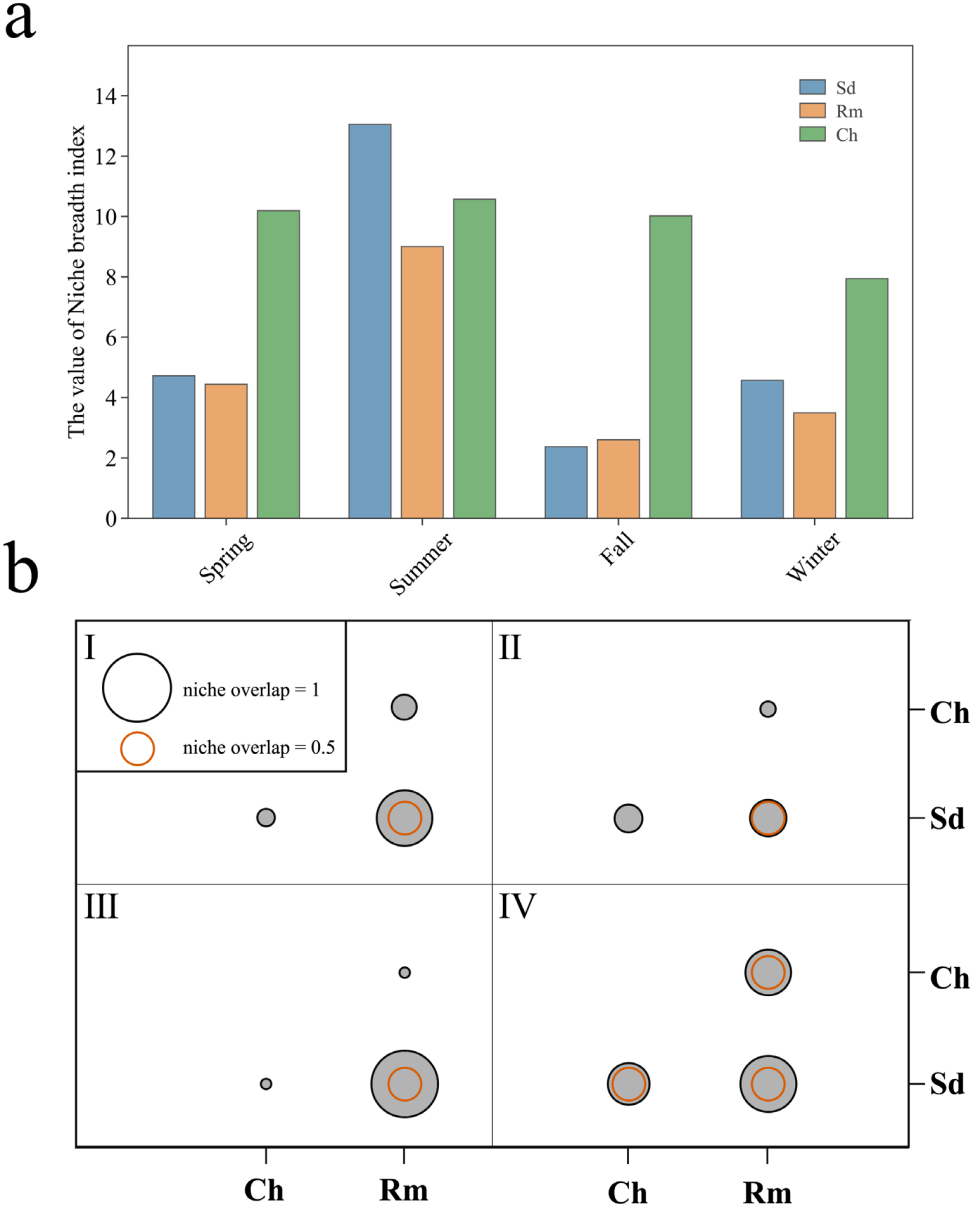
Niche overlap and breadth among three species based on four seasons. Larger circles depict higher niche overlap (I: spring, II: summer; III: fall, and IV: winter).

As an indicator of resource diversity utilization by organisms, niche breadth refers to the total range of resources that a species can utilize. A broader niche breadth indicates lower specialization and a tendency toward being a generalist. At the intra‐specific level, there were seasonal differences in the dietary niche breadth of sika deer and Reeves' muntjac, whereas there were no differences in Chinese hares. The dietary diversity and niche breadth value of sika deer and Reeve's muntjac were greatest during summer (Sd: Ba = 0.06; Rm: Ba = 0.04). At the interspecific level, sika deer exhibited higher dietary diversity and a broader niche breadth than Reeves' muntjac except during summer. In the fall, the niche overlap index between sika deer and Reeve's muntjac was the highest (*Q* = 0.98), indicating a nearly complete overlap in resource use. In winter and spring, the overlap index was 0.83 and 0.81, respectively, showing a significant niche overlap (*Q* > 0.6). Overall, the niche overlap index manifested as a higher value in sika deer versus Reeves' muntjac than in Chinese hares (Table 1; Figure 7).

**TABLE 1:** Niche breadth, trophic niche overlap, and dietary diversity (Shannon index) of three herbivores in TNNR.

	Trophic niche overlap among three species of TNNR											
	Spring			Summer			Fall			Winter		
	Sd	Rm	Ch	Sd	Rm	Ch	Sd	Rm	Ch	Sd	Rm	Ch
Sika deer	1.00	0.81	0.27	1.00	0.55	0.42	1.00	0.98	0.18	1.00	0.83	0.62
Reeves' muntjac		1.00	0.39		1.00	0.25		1.00	0.18		1.00	0.68
Chinese hare			1.00			1.00			1.00			1.00
Niche breadth (B)	4.72	4.44	10.19	13.05	9.00	10.57	2.37	2.60	10.02	4.57	3.49	7.94
Standardized niche breadth (Ba)	0.02	0.02	0.05	0.06	0.04	0.05	0.01	0.01	0.05	0.02	0.01	0.04
Dietary diversity	2.41	2.10	2.74	3.12	2.92	2.93	1.60	1.79	2.75	2.03	1.98	2.33

## 4 Discussion

The dietary spectra of herbivores are influenced by seasonal shifts and plant phenology. The moose ( *Alces alces* ) consumes leaves and branches annually (87.4%) and feeds on fallen leaves in late spring, whereas aquatic plants contribute to the diet in summer, with a primary reliance on leaves and branches in fall and winter (Yu and Xiao 1991). The diet of *Capreolus pygargus* varies seasonally, with a combination of grasses in spring, forbs in early summer, leafy green browses in late summer, and acorns and other fruits in fall (Wang, Feng, et al. 2023). These seasonal shifts in diet composition reflect the dynamic adaptation strategies of wildlife. The Japanese sika deer forages on preferred deciduous trees during summer and fall, while relying on nonpreferred plants in early winter through spring (Nakahama et al. 2021). In this study, sika deer displayed a preference for shrubs and herbaceous plants, with shrubs serving as the primary food item in all seasons, peaking in abundance during fall and followed by winter. Compared with herbs, shrubs contain higher levels of proteins and mineral elements, which serve as essential nutritional sources for maintaining dietary balance. Even in times of abundant resources, sika deer often fed on shrubs, reflecting their utilization of the “time minimization” and “energy maximization” foraging models (Guo et al. 2001). Some studies have indicated that Cervidae animals preferentially consume herbs (with high nutritional quality) during spring and summer, exhibiting reduced woody selectivity (with elevated cellulose content), whereas a higher preference for woody branches occurs during withering (Chen and Xiao 1991; Chen et al. 1998). In winter, Qingliangfeng sika deer primarily consumed dry and fallen leaves of shrubs and tree branches, as well as high‐sugar fruits such as *Crataegus cuneata* and *Smilax china* on the ground or hanging from branches (You 2009). Similarly, both sika deer and Reeves' muntjac exhibit increased consumption of woody plants in the TNNR, adopting a foraging strategy that maximized the winter benefits for sympatric species. In different seasons, the foraging patterns of Reeves' muntjac closely mirror those of sika deer. Yet, under conditions of resource scarcity, Reeves' muntjac increases their consumption of arbors and herbivores, while reducing their intake of shrubs. This may reflect a short‐term foraging strategy employed by sympatric species. In contrast, Chinese hares predominantly feed on herbaceous and shrubby plants, exhibiting seasonal variations. The consumption of arbors surpassed their browsing capacity (small size), which may be attributed to a short‐range opportunistic foraging strategy and even indirect intake (i.e., gnawing activities).

Tannins and their metabolites can exert toxic effects on animals, and many primates, such as *Colobus guereza*, *largely* avoid these compounds, which bind with proteins to reduce food digestibility (Fashing et al. 2007). However, alpine red deer ( *Cervus elaphus* ) show some selection for tannin‐ and fiber‐rich coniferous browse during the cold season, which could function as an adaptive mechanism during periods of resource scarcity, ensuring adequate biomass intake (Zweifel‐Schielly et al. 2012). Tannins also have been confirmed to be nontoxic to sika deer, as rumen microbes can ferment them (Li et al. 2013). In addition, tannins help prevent bloats in ruminants, reduce parasites to promote intestinal health (Molan et al. 2003), enhance food conversion efficiency (Clauss et al. 2003), and increase the reproductive and fawn survival rates of sika deer (Xing et al. 2023). In this study, Chinese fir, characterized by higher levels of lignin, cellulose, and secondary metabolites (i.e., tannin), was consumed by both sika deer and Reeves' muntjac despite abundant resource periods. This may be related to the requirement for tannins or influenced by the vast tracts of artificial Chinese fir trees, which have evolved as a long‐term adaptation.

Previous studies have shown that sika deer consume 153 plant species, exhibiting a preference for *Homonoia riparia* , *Fallopia multiflora* , *Lespedeza bicolor* , *Pueraria montana* , and *Vicia faba* based on traditional methods in TNNR (Yao et al. 2010). In contrast, this study identified a greater diversity of forage plants through the use of DNA metabarcoding. Compared to traditional methods, the development of DNA metabarcoding has provided precise detection of DNA remaining in fecal samples (Thongjued et al. 2024). Soininen et al. (2009) also demonstrated that DNA barcoding gave by far a taxonomically more detailed picture of small herbivores' diet than the microhistological analysis. Despite the DNA‐based molecular analysis facilitating unprecedented resolution in studying dietary diversity, these methods still face several challenges, issues such as the quality and quantity of DNA from fecal samples (Jedlicka et al. 2013), the universality of primers across species (especially in plants), as well as the variability in digestion efficiency among individual animals (Mackie 2002). Guo et al. (2018) pointed out a significant positive correlation between the actual herbage mass proportions in the experimental diets and the herbage‐DNA‐sequence proportions, which provided sufficiently favorable support for the further investigation of DNA barcoding for the quantification of plants in feces. Consequently, validating methods using captive animals may offer an approach to identify and limit bias in DNA metabarcoding‐based dietary analyses (Thongjued et al. 2024). Furthermore, employing multiple markers or primer sets can help limit primer and amplification biases in metabarcoding studies. Despite these advancements, after aligning sequences to both public and local DNA reference databases, the majority of reads were assigned to specific plant taxa. Still, some reads, particularly those with low abundance, remained unassigned, which could be attributed to the incompleteness of the reference databases. Establishing a comprehensive and standardized local reference database allows for more rapid, quality, and accurate species identification to effectively address this challenge. Therefore, to mitigate the limitations of representative sequences, new specimens from the TNNR should be continuously sampled for individual identification and sequencing (Shao et al. 2019).

The classic optimal foraging theory predicts that during seasons with abundant food resources, species display dietary specialization by prioritizing the consumption of the most profitable and nutritious resources, while exhibiting generalization by consuming broader forage (lower value ones) when resources are limited (Belovsky 1978). In the present study, sika deer and Reeves' muntjac occupied the broadest dietary niches in summer, reflecting a generalized foraging strategy encompassing a diverse range of food items within the reserve. Conversely, the dietary niche breadth was narrower in the fall. At this stage, the two species exhibited a stronger selectivity for *Rubus* spp., suggesting a trend toward dietary specialization. Subsequently, camera traps and field observations are required to further validate these findings. Moreover, we found that Chinese hares sustain a relatively large niche breadth, even during food‐scarce winters. It has been speculated that the Chinese hare, acting as an opportunistic feeder, maintains a broad dietary niche owing to its smaller body size and lower energy demand. Another important reason may involve the physicochemical conditions within the stomach and gut of the Chinese hare. Together with the structural complexity of plant cell walls and hard‐to‐digest food, the Chinese hare lacks the complex microbiota community compared to ruminant herbivores as a hindgut fermenter with a simple stomach, and faces inherent limitations in fully digesting all types of forage (Mackie 2002).

Niche overlap serves as a valuable metric for assessing levels of resource competition among species. A high degree of niche overlap is often interpreted as intense interspecific competition (Colwell and Futuyma 1971). Tibetan red deer share dietary habits comparable to those of other sympatric ungulates, inevitably resulting in interspecific conflict over resource utilization (Lv et al. 2020). Despite the observed niche overlap, spatiotemporal variations in the diet consumption between *Rhabdomys dilectus* and *Lophuromys acquilus* on Mount Kilimanjaro can serve as a resource‐partitioning mechanism that enables their coexistence (Mulungu et al. 2011; Thomas et al. 2022). Interspecific competition among sympatric species is primarily expressed as a compensation mechanism in niches; when species are similar in one dimension, they differ in another, and niche differentiation occurs along several dimensions to facilitate coexistence (Bagchi et al. 2003). Hence, differences in resource utilization can be used as a standard for judging niche partitioning (Xia et al. 2015). It is generally contented that large herbivores tend to broaden their niche breadth to avoid competition in low‐productive environments (Noor et al. 2013). For example, Red deer ( *Cervus elaphus* ) tend to increase their browsing intensity, whereas sika deer exhibit increase their bite diameter, reflecting short‐term foraging strategies by sharing similar forage with sympatric ungulates (Zhong et al. 2020). Similarly, other studies have shown that wild yak ( *Bos grunniens* ), Tibetan wild ass ( *Equus kiang* ), and Tibetan antelope (*Pantholops hodgsoni*) consume similar forage plants but varied greatly in their proportion (Shi et al. 2016). In terms of our study species, the summer niche overlap index was lowest between sika deer and Reeves' muntjac, suggesting a lower intensity of mutual interference in resource utilization, likely driven by high food diversity within the reserve (Zhang et al. 2021). Despite a high niche overlap (*Q* > 0.8) in other seasons, variations in the percentage of seasonal forage consumed by the two species might reflect the foraging strategies adopted in response to local adaptation and sympatric coexistence. In addition, the foraging positions (i.e., mature leaves, young leaves, branches, roots, and fruits; Zhou et al. 2018) and body size (Abraham et al. 2019) may also be an essential aspect that needs to be considered in follow‐up studies.

In conclusion, we demonstrated that sika deer and another two sympatric herbivores could adjust their food composition in response to seasonal variations in plant availability. Differences in the consumption and utilization of foraging plants produced niche partitioning, which further facilitated. Both sika deer and Reeves' muntjac are generalized herbivores, and Chinese hares are opportunistic feeders. *Rubus* spp. occupy a prominent position in the food spectrum of the two cervid species throughout the year, and biomass and dynamic monitoring of *Rubus* spp. plants need to be carried out and strengthened. Overall, DNA metabarcoding sequencing is appropriate for quantifying the diet of herbivores. This study highlights the seasonal diet and interspecific niche of three sympatric herbivores, which facilitate habitat improvements, artificial planting, and forage resource monitoring. However, research on feeding behavior by herbivores in relation to plant community cycles and seasonal nutritional value intake remains underexplored. To gain a comprehensive understanding of the feeding ecology and life history, future studies should consider the seasonal effects and nutritional value for the sympatric herbivores.

## Author Contributions

**Dandan Wang:** data curation (equal), formal analysis (equal), investigation (equal), methodology (equal), writing – original draft (lead). **Zhiming Cao:** data curation (equal), formal analysis (equal), investigation (equal), methodology (equal), writing – original draft (equal). **Yuqin Liu:** data curation (supporting), formal analysis (supporting), investigation (supporting), methodology (supporting), writing – original draft (supporting). **Ruofei Li:** data curation (supporting), formal analysis (supporting), investigation (supporting). **Ruitao Wu:** conceptualization (supporting), data curation (supporting), investigation (supporting). **Wenguo Wu:** data curation (equal), investigation (equal), methodology (equal). **Wuhua Liu:** data curation (equal), investigation (equal), methodology (equal). **Xiaolong Hu:** methodology (equal), project administration (equal), supervision (equal). **Yongtao Xu:** conceptualization (lead), data curation (lead), methodology (lead), project administration (lead), resources (lead), supervision (lead), visualization (lead), writing – review and editing (lead).

## Ethics Statement

No animals were captured, and fecal sample analyses were performed based on the noninvasive principle.

## Conflicts of Interest

The authors declare no conflicts of interest.

## Data Availability

Raw sequence data are archived in the NCBI Short Read Archive (http://www.ncbi.nlm.nih.gov/sra) as BioProject PRJNA1122487 and PRJNA1122507.

## Appendix A

**TABLE A1:** Species alpha diversity index in seasons (including, Chao1, Goods_coverage, Pielou_e, Shannon, and Simpson indices).

Sample	Seasons	Chao1 average	Pielou_e average	Shannon average	Simpson average	Goods_coverage average
Sika deer	Spring	599.7	0.42	3.34	0.77	0.9919
	Summer	523.36	0.41	3.2	0.76	0.9927
	Fall	412.21	0.29	2.19	0.52	0.9945
	Winter	266.47	0.26	1.85	0.49	0.9962
Reeves' muntjac	Spring	578.82	0.4	3.23	0.76	0.9922
	Summer	626.49	0.4	3.25	0.74	0.9915
	Fall	431.33	0.31	2.37	0.58	0.994
	Winter	302.95	0.31	2.24	0.56	0.9958
Chinese hare	Spring	407.15	0.32	2.44	0.64	0.9943
	Summer	372.05	0.33	2.46	0.65	0.9949
	Fall	338.99	0.33	2.38	0.61	0.9955
	Winter	274.37	0.36	2.39	0.64	0.9965

**TABLE A2:** Composition of food items consumed by sika deer in different seasons (RRA ≥ 1%).

Number	Species	Genus	Spring	Summer	Fall	Winter
			Total RRA percentage ≥ 1 (%)			
1	*Rubus* spp.	*Rubus*	31.58	20.44	61.01	51.77
2	*Loropetalum chinense*	*Loropetalum*	12.30	6.94	—	0.82
3	*Neolitsea aurata*	*Neolitsea*	0.58	14.01	4.57	—
4	*Eurya japonica*	*Eurya*	—	1.18	—	11.01
5	*Tripterygium wilfordii*	*Tripterygium*	—	1.62	7.48	—
6	*Ilex latifolia*	*Ilex*	5.91	3.07	—	—
7	*Euonymus grandiflorus*	*Euonymus*	—	—	—	6.10
8	*Pistacia chinensis*	*Pistacia*	2.26	2.44	1.01	—
9	*Rhododendron simsii*	*Rhododendron*	0.20	0.72	4.02	0.40
10	*Broussonetia papyrifera*	*Broussonetia*	0.23	3.16	1.14	0.59
11	*Maclura cochinchinensis*	*Maclura*	0.30	1.80	2.15	—
12	*Smilax china*	*Smilax*	1.99	0.73	—	1.26
13	*Quercus fabri*	*Quercus*	2.77	1.05	—	—
14	*Celtis sinensis*	*Celtis*	—	—	0.85	2.57
15	*Osmanthus fragrans*	*Osmanthus*	—	3.05	—	—
16	*Arrhenatherum elatius*	*Arrhenatherum*	—	—	—	3.02
17	*Cunninghamia lanceolata*	*Cunninghamia*	0.17	1.14	0.29	1.21
18	*Lysimachia congestiflora*	*Lysimachia*	0.84	—	1.00	0.81
19	*Pterocarya stenoptera*	*Pterocarya*	0.24	2.38	—	—
20	*Acorus calamus*	*Acorus*	—	2.61	—	—
21	*Nyssa sinensis*	*Nyssa*	—	2.06	0.50	—
22	*Sabia swinhoei*	*Sabia*	—	—	0.20	2.14
23	*Phyllostachys edulis*	*Phyllostachys*	—	0.60	0.27	1.31
24	*Oxalis corniculata*	*Oxalis*	0.28	—	0.62	0.79
25	*Maclura tricuspidata*	*Maclura*	—	—	—	1.66
26	*Paederia foetida*	*Paederia*	0.48	0.32	0.78	—
27	*Ligustrum lucidum*	*Ligustrum*	—	—	—	1.48
28	*Pueraria montana*	*Pueraria*	—	0.76	0.63	—
29	*Rosa rubus*	*Rosa*	0.46	0.42	0.44	—
30	*Camellia sinensis*	*Camellia*	—	—	1.26	—
31	*Vaccinium bracteatum*	*Vaccinium*	1.26	—	—	—
32	*Phyllanthus reticulatus*	*Phyllanthus*	—	—	1.13	—
33	*Prunus pseudocerasus*	*Prunus*	—	0.50	0.60	—

**TABLE A3:** Composition of food items consumed by Reeves' muntjac in different seasons (RRA ≥ 1%).

Number	Species	Genus	Spring	Summer	Fall	Winter
			Total RRA percentage ≥ 1 (%)			
1	*Smilax china*	*Smilax*	16.95	7.65	0.30	15.56
2	*Setaria viridis*	*Setaria*	3.95	1.93	23.05	1.56
3	*Poa annua*	*Poa*	11.23	10.81	2.14	—
4	*Rubus* spp.	*Rubus*	9.80	5.74	5.22	—
5	*Calamagrostis epigeios*	*Calamagrostis*	0.81	8.19	11.09	—
6	*Pueraria montana*	*Pueraria*	7.91	1.26	6.04	—
7	*Cunninghamia lanceolata*	*Cunninghamia*	—	—	6.87	4.47
8	*Lespedeza pilosa*	*Lespedeza*	2.65	8.30	—	—
9	*Rhus chinensis*	*Rhus*	—	—	—	10.64
10	*Phyllostachys edulis*	*Phyllostachys*	4.95	2.84	1.49	0.96
11	*Eleusine indica*	*Eleusine*	—	—	5.40	3.57
12	*Indigofera bungeana*	*Indigofera*	—	8.01	—	—
13	*Syringa oblata*	*Syringa*	6.19	—	1.16	—
14	*Scleromitrion diffusum*	*Scleromitrion*	0.20	0.21	0.19	5.02
15	*Dalbergia hupeana*	*Dalbergia*	—	—	5.53	—
16	*Dioscorea cirrhosa*	*Dioscorea*	—	5.34	—	—
17	*Oxalis corniculata*	*Oxalis*	1.66	0.54	0.13	2.31
18	*Neolitsea aurata*	*Neolitsea*	1.45	0.67	2.08	—
19	*Melia azedarach*	*Melia*	—	—	3.68	0.26
20	*Paederia foetida*	*Paederia*	2.31	0.63	0.71	—
21	*Miscanthus sinensis*	*Miscanthus*	—	0.47	0.61	2.55
22	*Lysimachia congestiflora*	*Lysimachia*	2.03	1.43	—	—
23	*Panicum bisulcatum*	*Panicum*	—	1.63	0.13	1.49
24	*Solidago canadensis*	*Solidago*	0.56	1.05	1.47	—
25	*Digitaria sanguinalis*	*Digitaria*	0.40	1.02	1.54	—
26	*Sedum bulbiferum*	*Sedum*	2.92	—	—	—
27	*Rhododendron simsii*	*Rhododendron*	—	2.60	—	0.29
28	*Chrysanthemum indicum*	*Chrysanthemum*	—	—	2.37	—
29	*Pinus massoniana*	*Pinus*	0.15	0.13	0.28	1.46
30	*Bidens pilosa*	*Bidens*	1.22	0.55	—	—
31	*Juniperus communis*	*Juniperus*	—	1.51	—	—
32	*Justicia quadrifaria*	*Justicia*	—	1.35	—	—
33	*Quercus fabri*	*Quercus*	—	—	1.24	—
34	*Secale cereale*	*Secale*	—	—	—	1.16
35	*Loropetalum chinense*	*Loropetalum*	0.16	—	0.38	0.49
36	*Polypogon fugax*	*Polypogon*	—	—	—	1.00

**TABLE A4:** Composition of food items consumed by Chinese hare in different seasons (RRA ≥ 1%).

Number	Species	Genus	Spring	Summer	Fall	Winter
			Total RRA percentage ≥ 1 (%)			
1	*Rubus* spp.	*Rubus*	15.20	10.75	63.95	36.42
2	*Loropetalum chinense*	*Loropetalum*	17.83	9.91	—	25.48
3	*Rhododendron simsii*	*Rhododendron*	0.59	8.94	6.46	1.45
4	*Pistacia chinensis*	*Pistacia*	9.30	5.20	1.46	—
5	*Eurya japonica*	*Eurya*	0.24	—	—	13.41
6	*Smilax china*	*Smilax*	1.24	11.79	—	—
7	*Oxalis corniculata*	*Oxalis*	4.18	3.13	1.01	0.12
8	*Cunninghamia lanceolata*	*Cunninghamia*	0.84	4.43	0.48	1.67
9	*Phyllostachys edulis*	*Phyllostachys*	0.20	1.50	1.41	2.46
10	*Camellia japonica*	*Camellia*	—	—	—	3.88
11	*Viola philippica*	*Viola*	—	3.60	—	—
12	*Euonymus grandiflorus*	*Euonymus*	—	—	—	2.94
13	*Quercus fabri*	*Quercus*	0.48	2.21	—	—
14	*Lespedeza pilosa*	*Lespedeza*	—	2.24	0.39	—
15	*Phyllanthus* sp.	*Phyllanthus*	—	2.03	0.45	—
16	*Vaccinium bracteatum*	*Vaccinium*	0.91	0.64	0.13	0.77
17	*Neolitsea aurata*	*Neolitsea*	0.69	0.72	1.00	—
18	*Boehmeria nivea*	*Boehmeria*	—	—	2.27	—
19	*Camellia sinensis*	*Camellia*	—	—	2.00	—
20	*Chrysanthemum indicum*	*Chrysanthemum*	—	—	1.91	—
21	*Maclura tricuspidata*	*Maclura*	—	—	—	1.88
22	*Pueraria montana*	*Pueraria*	—	1.14	0.72	—
23	*Lygodium japonicum*	*Lygodium*	0.35	0.37	1.04	—
24	*Prunus pseudocerasus*	*Prunus*	1.53	—	—	—
25	*Camellia oleifera*	*Camellia*	1.02	0.44	—	—
26	*Bidens pilosa*	*Bidens*	—	1.46	—	—
27	*Mallotus japonicus*	*Mallotus*	0.22	0.32	0.89	—
28	*Scleromitrion diffusum*	*Scleromitrion*	0.49	0.47	0.18	0.21
29	*Paederia foetida*	*Paederia*	0.21	—	1.09	—
30	*Acer buergerianum*	*Acer*	—	1.21	—	—
31	*Celtis sinensis*	*Celtis*	—	—	0.36	0.78
32	*Euscaphis japonica*	*Euscaphis*	0.26	0.88	—	—
33	*Phyllanthus reticulatus*	*Phyllanthus*	—	1.14	—	—

**Text A1 Construction of a barcode reference database**

To identify dietary plant sequences obtained from fecal samples, we constructed a DNA reference database of plants in TNNR (Reference list of potential forage plant species for herbivorous in TNNR). A total of 290 plant samples were collected in this study from TNNR. Plant DNA was extracted from green, dried leaf samples, with a total of 268 plant DNA samples and a yield rate of 94.4%. Ultimately, 162 high‐quality sequences were successfully obtained and submitted to Genbank using bankit (Genbank ID: PP081756–PP081917) and compiled into the local reference database of potential diets for sika deer. The length of these sequences ranged from 162 to 255 bp. We will continue to update our Taohongling references database with newly vouchered specimens.

The DNA extraction of the samples was performed using the Fore Gene Plant DNA Isolation Kit (Chengdu). The DNA optical density (OD) value was measured by an ultraviolet spectrophotometer, and the A260/A280 of most DNA extracts was between 1.70 and 2.21. The sequences for the trnL (UAA) gene universal primers were designated as c (5′‐CGAAATCGGTAGACGCTACG‐3′) and h (5’‐CCATTGAGTCTCTG CACCTATC‐3′). The PCR amplifications were carried out for primers c–h in a total volume of 25 μL containing 12.5 μL PCR mix, 1 μL DNA template, 1 μL each of forward and reverse primers, 9.5 μL ddH_2_O, and added PCR negative control (sterile water). The reaction conditions were as follows: denaturation at 94°C for 4 min, followed by 35 cycles at 94°C for 30 s, 56°C for 30 s, and 72°C for 45 s, with a final step of 10 min at 72°C. PCR products were subjected to quality evaluation using 1% agarose gel electrophoresis (200 V, 100 mA, 30 min). The bands with clear and qualified products were selected for further analysis and sent to Shanghai Saiheng Biological Technology Co. Ltd. for DNA fragment single‐end sequencing. The raw sequencing data were saved in FASTQ format.

## References

[ece371321-bib-0001] Abraham, J. O. , G. P. Hempson , and A. C. Staver . 2019. “Drought‐Response Strategies of Savanna Herbivores.” Ecology and Evolution 9, no. 12: 7047–7056. 10.1002/ece3.5270. 31380032 PMC6662422

[ece371321-bib-0002] Atwood, T. B. , S. A. Valentine , E. Hammill , et al. 2020. “Herbivores at the Highest Risk of Extinction Among Mammals, Birds, and Reptiles.” Science Advances 6, no. 32: eabb8458. 10.1126/sciadv.abb8458. 32923612 PMC7457337

[ece371321-bib-0003] Bagchi, S. , S. P. Goyal , and K. Sankar . 2003. “Niche Relationships of an Ungulate Assemblage in a Dry Tropical Forest.” Journal of Mammalogy 84, no. 3: 981–988. 10.1644/BBa-024.

[ece371321-bib-0004] Barco, A. , M. J. Raupach , S. Laakmann , H. Neumann , and T. Knebelsberger . 2016. “Identification of North Sea Molluscs With DNA Barcoding.” Molecular Ecology Resources 16, no. 1: 288–297. 10.1111/1755-0998.12440. 26095230

[ece371321-bib-0005] Belovsky, G. E. 1978. “Diet Optimization in a Generalist Herbivore: The Moose.” Theoretical Population Biology 14, no. 1: 105–134. 741393 10.1016/0040-5809(78)90007-2

[ece371321-bib-0006] Chao, A. 1984. “Nonparametric Estimation of the Number of Classes in a Population.” Scandinavian Journal of Statistics 11, no. 4: 265–270.

[ece371321-bib-0007] Chen, E. J. , T. P. Guan , and S. Li . 2022. “The Sex Ratio, Social Structure and Activity Pattern of Reeves' Muntjac (*Muntiacus reevesi*) in Minshan Mountains, Sichuan Province.” Acta Theriologica Sinica 42, no. 1: 1–11. 10.16829/j.slxb.150576.

[ece371321-bib-0008] Chen, H. P. , F. Li , Z. W. Sun , et al. 1998. “Seasonal Selection of Forages by Red Deer and Roe Deer in Relation to Availability and Quality of Forages in Mixed Forests, Tonghe, Northeastern China.” Biosphere Conservation: For Nature, Wildlife, and Humans 1, no. 2: 129–140. 10.20798/biospherecons.1.2_129.

[ece371321-bib-0009] Chen, H. P. , and Q. Z. Xiao . 1991. “Comparison of Winter Trophic Strategies Between Red Deer and Row Deer in Dailing Region.” Acta Ecologica Sinica 11, no. 4: 349–354.

[ece371321-bib-0010] Clauss, M. , K. Lason , J. Gehrke , et al. 2003. “Captive Roe Deer (*Capreolus capreolus*) Select for Low Amounts of Tannin Acid but Not Quebracho: Fluctuations of Preferences and Potential Benefits.” Comparative Biochemistry and Physiology, Part B: Biochemistry & Molecular Biology 136, no. 2: 369–382. 10.1016/s1096-4959(03)00244-6. 14529762

[ece371321-bib-0011] Codron, D. , J. Codron , J. A. Lee‐Thorp , et al. 2007. “Diets of Savanna Ungulates From Stable Carbon Isotope Composition of Faeces.” Journal of Zoology 273, no. 1: 21–29. 10.1111/j.1469-7998.2007.00292.x.

[ece371321-bib-0012] Colwell, R. K. , and D. J. Futuyma . 1971. “On the Measurement of Niche Breadth and Overlap.” Ecology 52, no. 4: 567–576. 10.2307/1934144. 28973805

[ece371321-bib-0013] Deagle, B. E. , A. C. Thomas , J. C. McInnes , et al. 2019. “Counting With DNA in Metabarcoding Studies: How Should We Convert Sequence Reads to Dietary Data?” Molecular Ecology 28, no. 2: 391–406. 10.1111/mec.14734. 29858539 PMC6905394

[ece371321-bib-0014] Edgar, R. C. 2018. “Accuracy of Taxonomy Prediction for 16S rRNA and Fungal ITS.” PeerJ 6: e4652. 10.7717/peerj.4652. 29682424 PMC5910792

[ece371321-bib-0015] Fashing, P. J. , E. S. Dierenfeld , and C. B. Mowry . 2007. “Influence of Plant and Soil Chemistry on Food Selection, Ranging Patterns, and Biomass of *Colobus guereza* in Kakamega Forest, Kenya.” International Journal of Primatology 28: 673–703. 10.1007/s10764-006-9096-2.

[ece371321-bib-0016] Fu, Y. , X. Jia , J. Hu , Y. Guo , and H. Zhu . 2006. “Summer Habitat Selection by Sika Deer in Taohongling Nature Reserve, Jiangxi Province.” Sichuan Journal of Zoology 25, no. 4: 863–865.

[ece371321-bib-0017] Gause, G. F. 2019. The Struggle for Existence: A Classic of Mathematical Biology and Ecology. Courier Dover Publications.

[ece371321-bib-0018] Good, I. J. 1953. “The Population Frequencies of Species and the Estimation of Population Parameters.” Biometrika 40, no. 3–4: 237–264. 10.1093/biomet/40.3-4.237.

[ece371321-bib-0019] Guo, Y. , H. Zhang , W. Chen , and Y. Zhang . 2018. “Herbivore‐Diet Analysis Based on Illumina MiSeq Sequencing: The Potential Use of an ITS2‐Barcoding Approach to Establish Qualitative and Quantitative Predictions of Diet Composition of Mongolian Sheep.” Journal of Agricultural and Food Chemistry 66, no. 37: 9858–9867. 10.1021/acs.jafc.8b02814. 30198261

[ece371321-bib-0020] Guo, Y. J. , R. J. Long , D. G. Zhang , and J. G. Chen . 2001. “The Seasonal Dynamics of Nutrient Contents in Some Dominant Shrubs and Forage Herbs on Alpine Meadow in Eastern Qilian Mountain, China.” Pratacultural Science 18, no. 6: 36–39.

[ece371321-bib-0021] Guo, Y. S. , and H. Z. Zheng . 2000. “On the Geological Distribution Taxonomic Status of Species and Evolution History of Sika Deer in China.” Acta Theriologica Since 20, no. 3: 168–179. 10.16829/j.slxb.2000.03.002.

[ece371321-bib-0022] Hambäck, P. A. , E. Weingartner , L. Dalén , H. Wirta , and T. Roslin . 2016. “Spatial Subsidies in Spider Diets Vary With Shoreline Structure: Complementary Evidence From Molecular Diet Analysis and Stable Isotopes.” Ecology and Evolution 6, no. 23: 8431–8439. 10.1002/ece3.2536. 28031795 PMC5167037

[ece371321-bib-0023] Hartvig, I. , A. G. Howe , E. N. B. Schmidt , C. Pertoldi , J. L. Nielsen , and R. M. Buttenschøn . 2021. “Diet of the European Bison ( *Bison bonasus* ) in a Forest Habitat Estimated by DNA Barcoding.” Mammal Research 66, no. 1: 123–136. 10.1007/s13364-020-00541-8.

[ece371321-bib-0024] Hou, J. J. , L. Li , Y. F. Wang , et al. 2021. “Influences of Submerged Plant Collapse on Diet Composition, Breadth, and Overlap Among Four Crane Species at Poyang Lake, China.” Frontiers in Zoology 18, no. 1: 1–17. 10.1186/s12983-021-00411-2. 34001190 PMC8130136

[ece371321-bib-0025] Hutchinson, M. C. , A. P. Dobson , and R. M. Pringle . 2022. “Dietary Abundance Distributions: Dominance and Diversity in Vertebrate Diets.” Ecology Letters 25, no. 4: 992–1008. 10.1111/ele.13948. 34967090

[ece371321-bib-0026] Jedlicka, J. A. , A. M. Sharma , and R. P. Almeida . 2013. “Molecular Tools Reveal Diets of Insectivorous Birds From Predator Fecal Matter.” Conservation Genetics Resources 5, no. 3: 879–885. 10.1007/s12686-013-9900-1.

[ece371321-bib-0027] Jiang, Z. G. 2009. Study on the Biodiversity of Taohongling Sika Deer National Nature Reserve in Jiangxi. Tsinghua University Press.

[ece371321-bib-0028] Johnson, J. S. , D. J. Spakowicz , B. Y. Hong , et al. 2019. “Evaluation of 16S rRNA Gene Sequencing for Species and Strain‐Level Microbiome Analysis.” Nature Communications 10, no. 1: 5029. 10.1038/s41467-019-13036-1. PMC6834636 31695033

[ece371321-bib-0029] Kartzinel, T. R. , P. A. Chen , T. C. Coverdale , et al. 2015. “DNA Metabarcoding Illuminates Dietary Niche Partitioning by African Large Herbivores.” Proceedings of the National Academy of Sciences of the United States of America 112, no. 26: 8019–8024. 10.1073/pnas.1503283112. 26034267 PMC4491742

[ece371321-bib-0030] Kartzinel, T. R. , and R. M. Pringle . 2020. “Multiple Dimensions of Dietary Diversity in Large Mammalian Herbivores.” Journal of Animal Ecology 89, no. 6: 1482–1496. 10.1111/1365-2656.13206. 32163591

[ece371321-bib-0031] Kearney, M. , S. J. Simpson , D. Raubenheimer , and B. Helmuth . 2010. “Modelling the Ecological Niche From Functional Traits.” Philosophical Transactions of the Royal Society, B: Biological Sciences 365, no. 1557: 3469–3483. 10.1098/rstb.2010.0034. PMC2981966 20921046

[ece371321-bib-0032] Kong, F. Q. , B. W. Shen , X. Y. Li , et al. 2024. “Analysis of Seasonal Activity Rhythm and Interspecific Differences in Sympatric Ungulates in Jiangxi Taohongling Reserve.” Chinese Journal of Wildlife 45: 242–250. 10.12375/ysdwxb.20240202.

[ece371321-bib-0033] Kowalczyk, R. , P. Taberlet , É. Coissac , et al. 2011. “Influence of Management Practices on Large Herbivore Diet‐Case of European Bison in Białowieża Primeval Forest (Poland).” Forest Ecology and Management 261, no. 4: 821–828. 10.1016/j.foreco.2010.11.026.

[ece371321-bib-0034] Kruskal, W. H. , and W. A. Wallis . 1952. “Use of Ranks in One‐Criterion Variance Analysis.” Journal of the American Statistical Association 47, no. 260: 583–621. 10.1080/01621459.1952.10483441.

[ece371321-bib-0035] Lamichhane, S. , B. Shrestha , B. P. C. Tharu , et al. 2025. “Narrow Dietary Niche With High Overlap Between Snow Leopards and Himalayan Wolves Indicates Potential for Resource Competition in Shey Phoksundo National Park, Nepal.” Ecology and Evolution 15, no. 1: e70873. 10.1002/ece3.70873. 39844787 PMC11751241

[ece371321-bib-0036] Lei, W. X. , W. Wei , D. Pu , et al. 2024. “Comparative Analysis of Trophic Niche Using Stable Isotopes Provides Insight Into Resource Use of Giant Pandas.” Integrative Zoology 19, no. 6: 1151–1162. 10.1111/1749-4877.12765. 37814789

[ece371321-bib-0037] Levins, R. 1968. Evolution in Changing Environments. Princeton University Press.

[ece371321-bib-0038] Li, R. , D. Wang , Z. Cao , et al. 2024. “DNA Metabarcoding Reveals Diet Diversity and Niche Partitioning by Two Sympatric Herbivores in Summer.” PeerJ 12: e18665. 10.7717/peerj.18665. 39726746 PMC11670756

[ece371321-bib-0039] Li, Z. P. , H. L. Liu , G. Y. Li , et al. 2013. “Molecular Diversity of Rumen Bacterial Communities From Tannin‐Rich and Fiber‐Rich Forage Fed Domestic Sika Deer (*Cervus nippon*) in China.” BMC Microbiology 13: 151. 10.1186/1471-2180-13-151. 23834656 PMC3723558

[ece371321-bib-0040] Lin, L. , Z. Guo , J. Bai , Z. Zhong , J. Li , and Q. Guo . 2024. “Seasonal Covariations of Diet‐Gut Microbiota in the Adaptation of the Newly Reintroduced Père David's Deer ( *Elaphurus davidianus* ) to the Northern Habitat.” Global Ecology and Conservation 52: e02983. 10.1016/j.gecco.2024.e02983.

[ece371321-bib-0041] Lv, Z. H. , W. Q. Zhang , H. Liu , M. H. Zhang , and Y. R. Li . 2020. “Comparison on Feeding Habits of Cervus Wallichii and Sympatric Ungulates and Domestic Animals in Green Grass Period.” Chinese Journal of Applied Ecology 31, no. 2: 651–658. 10.13287/j.1001-9332.202002.002. 32476360

[ece371321-bib-0042] Mackie, R. I. 2002. “Mutualistic Fermentative Digestion in the Gastrointestinal Tract: Diversity and Evolution.” Integrative and Comparative Biology 42, no. 2: 319–326. 10.1093/icb/42.2.319. 21708724

[ece371321-bib-0043] Metzger, C. , S. Ursenbacher , and P. Christe . 2009. “Testing the Competitive Exclusion Principle Using Various Niche Parameters in a Native (*Natrix maura*) and an Introduced (*N. tessellata*) Colubrid.” Amphibia‐Reptilia 30, no. 4: 523–531. 10.1163/156853809789647031.

[ece371321-bib-0044] Molan, A. L. , A. J. Duncan , T. N. Barry , and W. C. McNabb . 2003. “Effects of Condensed Tannins and Crude Sesquiterpene Lactones Extracted From Chicory on the Motility of Larvae of Deer Lungworm and Gastrointestinal Nematodes.” Parasitology International 52, no. 3: 209–218. 10.1016/s1383-5769(03)00011-4. 14550476

[ece371321-bib-0045] Mulungu, L. S. , T. A. Mahlaba , A. W. Massawe , et al. 2011. “Dietary Differences of the Multimammate Mouse, *Mastomys natalensis* (Smith, 1834), Across Different Habitats and Seasons in Tanzania and Swaziland.” Wildlife Research 38, no. 7: 640–646. 10.1071/wr11028.

[ece371321-bib-0046] Nakahama, N. , T. Furuta , H. Ando , S. Setsuko , A. Takayanagi , and Y. Isagi . 2021. “DNA Meta‐Barcoding Revealed That Sika Deer Foraging Strategies Vary With Season in a Forest With Degraded Understory Vegetation.” Forest Ecology and Management 484: 118637. 10.1016/j.foreco.2020.118637.

[ece371321-bib-0047] Noor, A. , B. Habib , and S. Kumar . 2013. “Habitat Selection and Niche Segregation Between Chital and Nilgai in Keoladeo National Park, India.” European Journal Zoological Research 2, no. 2: 1–9. http://scholarsresearchlibrary.com/archive.html.

[ece371321-bib-0048] Owen‐Smith, N. , J. M. Fryxell , and E. H. Merrill . 2010. “Foraging Theory Upscaled: The Behavioural Ecology of Herbivore Movement.” Philosophical Transactions of the Royal Society, B: Biological Sciences 365, no. 1550: 2267–2278. 10.1098/rstb.2010.0095. PMC2894968 20566503

[ece371321-bib-0049] Pianka, E. R. 1973. “The Structure of Lizard Communities.” Annual Review of Ecology and Systematics 4, no. 1: 53–74.

[ece371321-bib-0050] Pompanon, F. , B. E. Deagle , W. O. Symondson , D. S. Brown , S. N. Jarman , and P. Taberlet . 2012. “Who Is Eating What: Diet Assessment Using Next Generation Sequencing.” Molecular Ecology 21, no. 8: 1931–1950. 10.1111/j.1365-294x.2011.05403.x. 22171763

[ece371321-bib-0051] Prins, H. H. , D. W. F. Boer , H. V. Oeveren , A. Correia , J. Mafuca , and H. Olff . 2006. “Co‐Existence and Niche Segregation of Three Small Bovid Species in Southern Mozambique.” African Journal of Ecology 44, no. 2: 186–198. 10.1111/j.1365-2028.2006.00619.x.

[ece371321-bib-0052] Ratkiewicz, M. , E. Coissac , M. Świsłocka , et al. 2024. “Winter Diet Overlap Among Moose, Roe Deer and Red Deer in Coniferous and Mixed Forests Depends on Snow Cover Depth.” Forest Ecology and Management 556: 121710. 10.1016/j.foreco.2024.121710.

[ece371321-bib-0053] Rognes, T. , T. Flouri , B. Nichols , C. Quince , and F. Mahé . 2016. “Vsearch: A Versatile Open Source Tool for Metagenomics.” PeerJ 4: e2584. 10.7717/peerj.2584. 27781170 PMC5075697

[ece371321-bib-0054] Schaller, G. B. 2000. Wildlife of the Tibetan Steppe. University of Chicago Press.

[ece371321-bib-0055] Schloss, P. D. , D. Gevers , and S. L. Westcott . 2011. “Reducing the Effects of PCR Amplification and Sequencing Artifacts on 16S rRNA‐Based Studies.” PLoS One 6, no. 12: e27310. 10.1371/journal.pone.0027310. 22194782 PMC3237409

[ece371321-bib-0056] Shannon, C. E. 1948. “A Mathematical Theory of Communication.” Bell System Technical Journal 27, no. 3: 379–423. 10.1145/584091.584093.

[ece371321-bib-0057] Shao, X. N. , D. Z. Song , Q. W. Huang , S. Li , and M. Yao . 2019. “Fast Surveys and Molecular Diet Analysis of Carnivores Based on Fecal DNA and Metabarcoding.” Biodiversity Science 27, no. 5: 543–556. 10.17520/biods.2018214.

[ece371321-bib-0058] Shi, J. B. , F. Lu , X. W. Li , et al. 2016. “Dietary Overlap and Co‐Existence of Sympatric Wild Yak, Tibetan Wild Ass and Tibetan Antelope in Arjin Shan National Nature Reserve, Xinjiang Province, China.” Wildlife Research 43, no. 4: 323–331. 10.1071/wr16045.

[ece371321-bib-0059] Simpson, E. H. 1949. “Measurement of Diversity.” Nature 163: 688. 10.1038/163688a0.

[ece371321-bib-0060] Soininen, E. M. , A. Valentini , E. Coissac , et al. 2009. “Analysing Diet of Small Herbivores: The Efficiency of DNA Barcoding Coupled With High‐Throughput Pyrosequencing for Deciphering the Composition of Complex Plant Mixtures.” Frontiers in Zoology 6: 1–9. 10.1186/1742-9994-6-16. 19695081 PMC2736939

[ece371321-bib-0088] Srilopan, S. , D. Lewanzik , S. Bumrungsri , et al. 2025. “Large and high‐altitude foraging ranges suggests importance of Wrinkle‐lipped free‐tailed bats (*Mops plicatus*) for consuming dispersing pest insects.” Oecologia 207, no. 2. 10.1007/s00442-025-05671-x. 39921762

[ece371321-bib-0061] Su, T. , G. Cui , Z. Man , et al. 2023. “Interspecific Association of Sika Deer in Terrestrial Animal Communities of Liancheng National Nature Reserve, China.” Integrative Zoology 18, no. 4: 688–703. 10.1111/1749-4877.12700. 36549005

[ece371321-bib-0062] Taberlet, P. , E. Coissac , F. Pompanon , C. Brochmann , and E. Willerslev . 2012. “Towards Next‐Generation Biodiversity Assessment Using DNA Metabarcoding.” Molecular Ecology 21, no. 8: 2045–2050. 10.1111/j.1365-294x.2012.05470.x. 22486824

[ece371321-bib-0063] Thomas, S. M. , G. E. Soka , L. S. Mulungu , and F. B. Makonda . 2022. “Spatial‐Temporal Variations in Dietary Consumption of Two Dominant Rodent Species (*Rhabdomys dilectus* and *Lophuromys acquilus*) on Mount Kilimanjaro, Tanzania.” Diversity 14, no. 8: 659. 10.3390/d14080659.

[ece371321-bib-0064] Thongjued, K. , K. Garcia , D. Scott , D. J. Gonthier , and J. R. Dupuis . 2024. “DNA Metabarcoding Diet Analysis in a Generalist Omnivore: Feeding Trials Reveal the Efficacy of Extraction Kits and a Multi‐Locus Approach for Identifying Diverse Diets.” Integrative Zoology 19, no. 5: 790–806. 10.1111/1749-4877.12806. 38297429

[ece371321-bib-0065] Tinker, M. T. , G. Bentall , and J. A. Estes . 2008. “Food Limitation Leads to Behavioral Diversification and Dietary Specialization in Sea Otters.” Proceedings of the National Academy of Sciences of the United States of America 105: 560–565. 10.1073/pnas.0709263105. 18195370 PMC2206575

[ece371321-bib-0066] Valentini, A. , F. Pompanon , and P. Taberlet . 2009. “DNA Barcoding for Ecologists.” Trends in Ecology & Evolution 24, no. 2: 110–117. 10.1016/j.tree.2008.09.011. 19100655

[ece371321-bib-0067] Wallace, R. 1981. “An Assessment of Diet‐Overlap Indexes.” Transactions of the American Fisheries Society 110, no. 1: 72–76. 10.1577/1548-8659(1981)110<72:AAODI>2.0.CO;2.

[ece371321-bib-0068] Wang, D. D. , X. L. Hu , M. L. Li , et al. 2023. “Diet Composition and Interspecific Niche of Taohongling Sika Deer (*Cervus nippon kopschi*) and Its Sympatric Reeve's Muntjac (*Muntiacus reevesi*) and Chinese Hare (*Lepus sinensis*) in Winter (Animalia, Mammalia).” ZooKeys 1149: 17–36. 10.3897/zookeys.1149.96936. 37234447 PMC10208087

[ece371321-bib-0069] Wang, L. , J. Feng , P. Mou , M. Pu , and T. M. Wang . 2023. “Relative Abundance of Roe Deer (*Capreolus pygargus*) Related to Overstory Structure and Understory Food Resources in Northeast China.” Global Ecology and Conservation 46: e02542. 10.1016/j.gecco.2023.e02542.

[ece371321-bib-0070] Wang, X. F. , Y. F. Zhong , J. W. Zhan , et al. 2021. “Analysis on Activity Rhythm of *Lophura nycthemera* Population in Taohongling Sika Deer National Nature Reserve Based on Infrared Camera Technology.” Modern Agricultural Science and Technology 19: 182–184. 10.3969/j.issn.1007-5739.2021.19.067.

[ece371321-bib-0071] Wei, F. W. 2022. Taxonomy and Distribution of Mammals in China. China Science Publish & Media.

[ece371321-bib-0072] Whitehead, G. K. 1972. Deer of the World, 194. Constable and CompanyLtd.

[ece371321-bib-0073] Whittaker, R. H. 1972. “Evolution and Measurement of Species Diversity.” Taxon 21, no. 2–3: 213–251. 10.2307/1218190.

[ece371321-bib-0074] Xia, J. , F. Wu , W. Z. Hu , J. L. Fang , and X. J. Yang . 2015. “The Coexistence of Seven Sympatric Fulvettas in Ailao Mountains, Ejia Town, Yunnan Province.” Zoological Research 36, no. 1: 18–28. 10.13918/j.issn.2095-8137.2015.1.18. 25730457 PMC4821172

[ece371321-bib-0075] Xiao, Y. , R. J. Jiang , R. Yin , et al. 2023. “Trophic Niche and Interspecific Relationship of Five Eels in the Waters of the Zhoushan Islands.” Journal of Fisheries of China 47, no. 7: 079306. 10.11964/jfc.20220113278.

[ece371321-bib-0076] Xing, X. M. , C. Ai , T. J. Wang , et al. 2023. “The First High‐Quality Reference Genome of Sika Deer Provides Insights for High‐Tannin Adaptation.” Genomics, Proteomics & Bioinformatics 21, no. 1: 203–215. 10.1016/J.GPB.2022.05.008. PMC10372904 35718271

[ece371321-bib-0077] Xiong, M. , X. Shao , Y. Long , et al. 2016. “Molecular Analysis of Vertebrates and Plants in Scats of Leopard Cats ( *Prionailurus bengalensis* ) in Southwest China.” Journal of Mammalogy 97, no. 4: 1054–1064. 10.1093/jmammal/gyw061.

[ece371321-bib-0078] Xu, Y. T. , D. D. Wang , X. L. Hu , et al. 2024. “Summer Diet of South China Sika Deer (*Cervus nippon kopschi*) in Taohongling Based on High‐Throughput Sequencing and Metabarcoding trnL.” Pakistan Journal of Zoology 56, no. 2: 771–780. 10.17582/journal.pjz/20220629000640.

[ece371321-bib-0079] Yao, H. , P. Wang , G. Davison , et al. 2021. “How Do Snow Partridge (*Lerwa lerwa*) and Tibetan Snowcock (*Tetraogallus tibetanus*) Coexist in Sympatry Under High‐Elevation Conditions on the Qinghai‐Tibetan Plateau?” Ecology and Evolution 11, no. 24: 18331–18341. 10.1002/ece3.8424. 35003676 PMC8717327

[ece371321-bib-0080] Yao, Z. S. , X. R. Xu , and P. Xu . 2010. The Analysis of Food Plants of Sika Deer in Taohongling Nature Reserve, Jiangxi Province. Proceedings and Abstracts of the 9th National Symposium on Natural Medicinal Material Resources, Guangzhou, China.

[ece371321-bib-0081] You, W. Y. 2009. Study on the Forage Resource of South China Sika Deer in Qingliangfeng Natural Reserve (Master Thesis). Zhejiang Forestry University.

[ece371321-bib-0082] Yu, X. C. , and Q. Z. Xiao . 1991. “The Food Composition and Seasonal Change of the Moose in HeiHe Forest Area.” Acta Theriologica Since 11, no. 4: 258. 10.16829/j.slxb.1991.04.004.

[ece371321-bib-0083] Zhang, K. C. , Q. H. Zhou , H. L. Xu , and Z. H. Huang . 2021. “Diet, Food Availability, and Climatic Factors Drive Ranging Behavior in White‐Headed Langurs in the Limestone Forests of Guangxi, Southwest China.” Zoological Research 42, no. 4: 406–411. 10.24272/j.issn.2095-8137.2020.292. 34075733 PMC8317190

[ece371321-bib-0084] Zhang, Q. J. , B. Yang , Q. Fu , L. Wang , X. Gong , and Y. B. Zhang . 2020. “The Winter Diet of Sambar (*Rusa unicolor*) in the Qionglai Mountains.” Biodiversity Science 28, no. 10: 1192–1201. 10.17520/biods.2020063.

[ece371321-bib-0085] Zhong, L. Q. , X. L. Zhi , Y. Sun , et al. 2020. “Winter Foraging of Sympatric Red Deer and Sika Deer in Northeast China: Diet Composition, Forage Selection, Bite Diameter and Browse Intensity.” Journal of Forest Research 25, no. 4: 276–284. 10.1080/13416979.2020.1762025.

[ece371321-bib-0086] Zhou, Q. H. , Z. H. Huang , H. Wei , and C. M. Huang . 2018. “Variations in Diet Composition of Sympatric *Trachypithecus francoisi* and *Macaca assamensis* in the Limestone Habitats of Nonggang, China.” Zoological Research 39, no. 4: 284–290. 10.24272/j.issn.2095-8137.2018.046. 29616679 PMC5968857

[ece371321-bib-0087] Zweifel‐Schielly, B. , Y. Leuenberger , M. Kreuzer , and W. Suter . 2012. “A Herbivore's Food Landscape: Seasonal Dynamics and Nutritional Implications of Diet Selection by a Red Deer Population in Contrasting Alpine Habitats.” Journal of Zoology 286, no. 1: 68–80. 10.1111/j.1469-7998.2011.00853.x.

